# Dlk1-Dio3 cluster miRNAs regulate mitochondrial functions in the dystrophic muscle in Duchenne muscular dystrophy

**DOI:** 10.26508/lsa.202201506

**Published:** 2022-10-20

**Authors:** Ai Vu Hong, Nathalie Bourg, Peggy Sanatine, Jerome Poupiot, Karine Charton, Evelyne Gicquel, Emmanuelle Massourides, Marco Spinazzi, Isabelle Richard, David Israeli

**Affiliations:** 1 Genethon, Evry, France; 2 Université Paris-Saclay, Univ Evry, Inserm, Généthon, Integrare Research Unit UMR_S951, Evry, France; 3 CECS, I-STEM, UEVE INSERM UMR861, AFM, Corbeil-Essonnes, France; 4 Neuromuscular Reference Center, Department of Neurology, CHU d’Angers, Angers, France; 5 Institute of Neurobiology and Neuropathology CHU d’Angers, Angers, France

## Abstract

Duchenne muscular dystrophy (DMD) is a severe muscle disease caused by impaired expression of dystrophin. Whereas mitochondrial dysfunction is thought to play an important role in DMD, the mechanism of this dysfunction remains to be clarified. Here we demonstrate that in DMD and other muscular dystrophies, a large number of Dlk1-Dio3 clustered miRNAs (DD-miRNAs) are coordinately up-regulated in regenerating myofibers and in the serum. To characterize the biological effect of this dysregulation, 14 DD-miRNAs were simultaneously overexpressed in vivo in mouse muscle. Transcriptomic analysis revealed highly similar changes between the muscle ectopically overexpressing 14 DD-miRNAs and the mdx diaphragm, with naturally up-regulated DD-miRNAs. Among the commonly dysregulated pathway we found repressed mitochondrial metabolism, and oxidative phosphorylation (OxPhos) in particular. Knocking down the DD-miRNAs in iPS-derived skeletal myotubes resulted in increased OxPhos activities. The data suggest that (1) DD-miRNAs are important mediators of dystrophic changes in DMD muscle, (2) mitochondrial metabolism and OxPhos in particular are targeted in DMD by coordinately up-regulated DD-miRNAs. These findings provide insight into the mechanism of mitochondrial dysfunction in muscular dystrophy.

## Abstract (precis)

A large number of DLK1-DIO3 miRNAs are up-regulated in the muscles and the serum of Duchenne muscular dystrophy, animal models, and patients. Mitochondrial functions, and in particular oxidative phosphorylation, are targeted by these coordinately up-regulated DLK1-DIO3 miRNAs.

## Graphical Abstract

**Figure:**
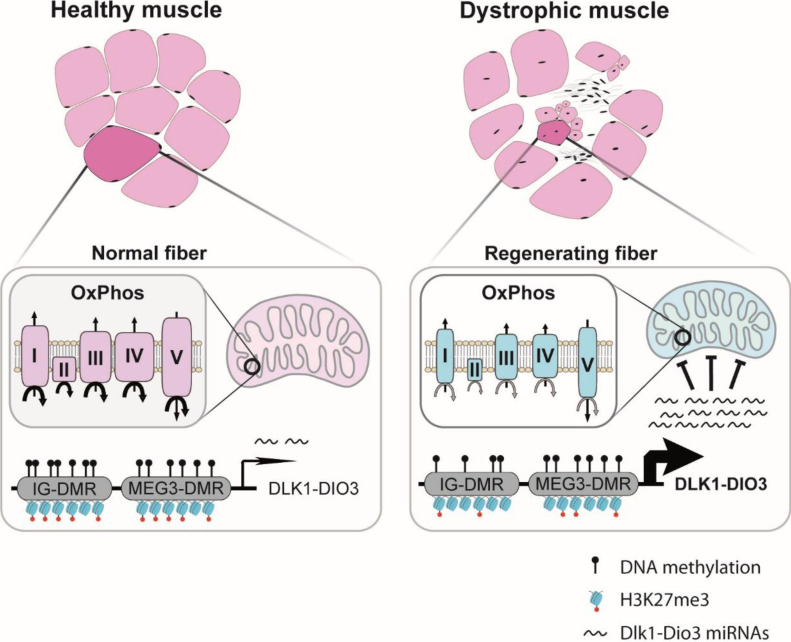

## Introduction

Duchenne muscular dystrophy (DMD) is an X-linked severe progressive muscle disease caused by mutations in the *Dmd* gene that codes for the dystrophin protein. The disease severely affects the motor function and leads to the premature death of the patient, primarily because of respiratory and cardiac failures (Duan et al, 2021). In striated muscle, dystrophin and its associated proteins link the intracellular cytoskeletal network and the extracellular matrix, to stabilize the myofibers. The lack of dystrophin promotes a pathological cascade, which includes structural defect in the sarcolemma, calcium overload, hyperactive proteases, oxidative stress, mitochondrial dysfunction, chronic inflammation, muscle degeneration, and impaired regenerative capacity, and in advanced stage the replacement of the contractile tissue by fibrotic and fat tissues (Duan et al, 2021; Ohlendieck & Swandulla, 2021). Initial attempts for the characterization of miRNA perturbations in DMD resulted in identification of dysregulation of the muscle-enriched myomiRs, including miR-1, miR-206 and miR-133 (Cacchiarelli et al, 2011; Roberts et al, 2012; Vignier et al, 2013; Zaharieva et al, 2013). Soon after, in a study of the GRMD dog model for DMD, we identified the dysregulation of cardiac-enriched miRNA, including miR-208a/b and miR-499, and of many miRNAs originated from Dlk1-Dio3 locus (Jeanson-Leh et al, 2014). Dysregulation in DMD of Dlk1-Dio3 miRNAs was confirmed in two additional investigations (Sanson et al, 2020; Amor et al, 2021).

The DLK1-Dio3 imprinted locus, which is located on chromosome 14q22 and 12qF1 (human/mouse, respectively), contains paternally expressed protein-coding genes and maternally expressed noncoding RNAs. The paternally protein-coding genes are the *Dlk1*, *Rtl1* and *Dio3*. The maternally noncoding RNAs are the *MEG3*/*Gtl2*, *MEG8*/*Rian*, and *MEG9*/*Mirg* (human/mouse nomenclature, respectively), and the largest miRNA mega-cluster in the human genome (Seitz et al, 2004), referred to us as DD-miRNAs. The Dlk1-Dio3 genomic locus, which is highly conserved in mammalians (da Rocha et al, 2008), is involved in human in a wide range of developmental processes and pathological conditions (Benetatos et al, 2013; Ioannides et al, 2014; Ogata & Kagami, 2016). The DD-miRNAs mega-cluster was shown to play a critical role in fetal development and postnatal growth (da Rocha et al, 2008; Ioannides et al, 2014; Ogata & Kagami, 2016). Initial indications for Dlk1-Dio3 locus involvement in the muscular system came from the identification of the muscular hypertrophy Callipyge phenotype in the sheep (Charlier et al, 2001). DD-miRNA were later shown to modulate the activity of skeletal (Snyder et al, 2013; Castel et al, 2018; Wüst et al, 2018), and cardiac (Dill & Naya, 2018) muscles. However, the biological functions of these miRNAs in the context of muscular dystrophy remains relatively unexplored.

In the present study, we showed that the DD-miRNA dysregulation is associated to muscle regeneration since, in addition to DMD, it was found also in several mouse models for other muscular dystrophies, as well as in regenerating normal muscle. We used the mdx mouse model for the investigation of DD-miRNAs expression control and biological functions in DMD. Combined analysis of DD-miRNAs target prediction, and of the transcriptome of dystrophic muscles, suggested that DD-miRNAs may target mitochondrial metabolism, and particularly the oxidative phosphorylation (OxPhos) system. Indeed, in vivo overexpression of 14 selected DD-miRNAs, in healthy muscles, drastically reduced mitochondrial OxPhos, and importantly, partly resembled the transcriptome of the dystrophic muscle. Furthermore, knocking down the entire DD-miRNAs cluster in hiPSC-derived myotubes resulted in increased RNA and proteins expression of OxPhos components, and the increased activity of the mitochondrial 1-5 complexes. Our data suggest a cooperative regulation by DD-miRNAs of mitochondrial functions in DMD and possibly in other situations of muscle regeneration.

## Results

### Up-regulation of DD-miRNA in regenerating myofibers and in the serum is associated to muscle regeneration

**Figure 1.**
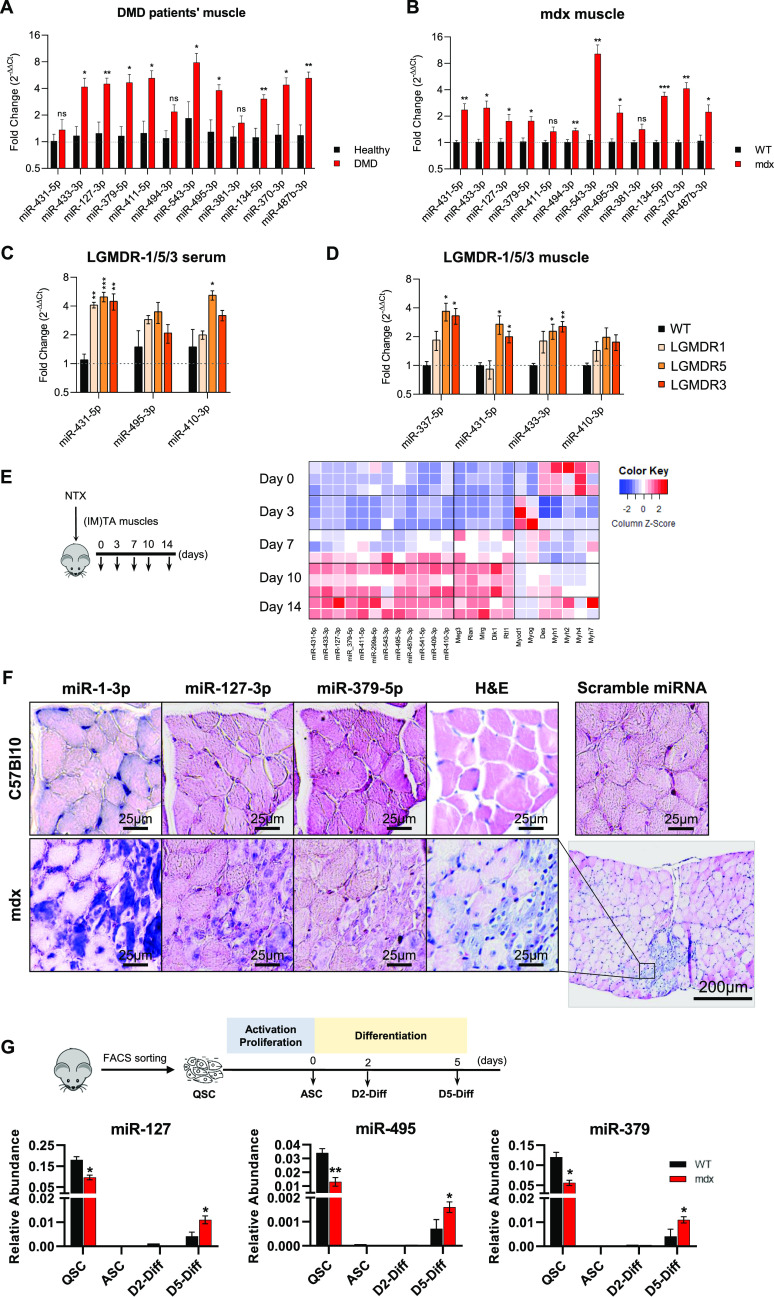
Characterization of DD-miRNAs dysregulation in the regenerating muscle. **(A)** Relative levels of DD-miRNAs in muscle biopsies of Duchenne muscular dystrophy patients compared with healthy controls (n = 4, the Duchenne muscular dystrophy cohort is described in the extended materials and methods supplemental data). **(B)** Relative expression levels of DD-miRNAs in diaphragm muscle of mdx mouse model compared with C57Bl10 control (5-week old, n = 6). **(C, D)** Relative expression levels of DD-miRNAs in the serum (C) and TA muscle (D) of mouse models for LGMDR1 (calpainopathy), LGMDR3 (α-sarcoglycanopathy), and LGMDR5 (γ-sarcoglycanopathy), compared with C57Bl6 control (5-wk-old n = 4). **(E)** The heat map presents the expression level of DD-miRNAs, of other transcripts of the Dlk1-Dio3 locus, and of myogenic transcription factors, during in vivo muscle regeneration in the model of notexin-induced damage (TA muscle, n = 2–3, RT-qPCR expression data were normalized to the average of U6 and miR-93-5p). Relative expression levels are illustrated by column Z-scores, colored from blue to red, indicating from lowest to highest expression. **(F)** ISH of miR-1-3p, miR-127-3p, and miR-379-5p in diaphragm of mdx and controls (Scale bar: 25 *µ*m). Nucleus was colored in pink, whereas miRNAs of interest was colored in dark blue. Scramble probes served as negative control. Regenerated myofibers are visible in the H&E staining (Scale bar: 200 *µ*m). **(G)** Relative expression of three DD-miRNAs through in vitro differentiation of satellite cells from muscles of 5-wk-old mdx and C57Bl10 control mice (n = 3). QSC, quiescent satellite cells; ASC, activated satellite cells; D2/D5-Diff, Day 2/5 differentiated myotubes; IM, intramuscular injection. Data in all graphical presentations are presented as mean ± SEM. Statistics were performed with *t* test. *P < 0.05; **P < 0.01; ***P < 0.001.

In previous studies, we identified a dysregulation of a large number of miRNAs of the Dlk1-Dio3 cluster (DD-miRNAs) in the serum of the GRMD dog, a model for DMD (Jeanson-Leh et al, 2014), and in the plasma of DMD patients (Amor et al, 2021). In the GRMD model, we found that DD-miRNAs are among the most highly up-regulated miRNA, not only in the circulation, but also in the muscle (Sanson et al, 2020). In the present study, we confirmed this observation across species. In particular, we quantified DD-miRNAs in muscle biopsies of DMD patients (Fig 1A, n = 4), and mdx mouse. In the mdx mouse, we analyzed the diaphragm muscle, presenting similar phenotype to human DMD skeletal muscles (Stedman et al, 1991) (Fig 1B, 5-wk-old mice, n = 6) and confirmed their up-regulation in the dystrophic muscle. Thus, DD-miRNAs are up-regulated in DMD in the muscles (in the mdx and GRMD models) and in the serum (in the GRMD model and in human patients).

To define whether these dysregulations are specific to DMD, we quantified DD-miRNAs in serum and muscle biopsies of a collection of mouse models for limb-girdle muscular dystrophies, including LGMDR1 (calpainopathy), LGMDR5 (γ sarcoglycanopathy), and LGMDR3 (α sarcoglycanopathy). Interestingly, up-regulation of DD-miRNAs was identified in both serum and the Tiblais Anterior muscle of these disease models (5-wk-old mice, n = 4, Fig 1C and D). DD-miRNAs are therefore expressed in the healthy muscle and up-regulated further in distinct muscular dystrophies. Next, we analyzed the expression of DD-miRNAs during muscle regeneration in the model of myotoxin-induced injury. The levels of a collection of DD-miRNAs, along with other maternal and paternal transcripts from the same locus (*Meg3*, *Rian*, *Mirg*, *Dlk1*, and *Rtl1*), increased gradually from day 7 to 14 post-injury, confirming up-regulation in the regenerating muscle independently of a genetic defect (Fig 1E).

**Figure S1.**
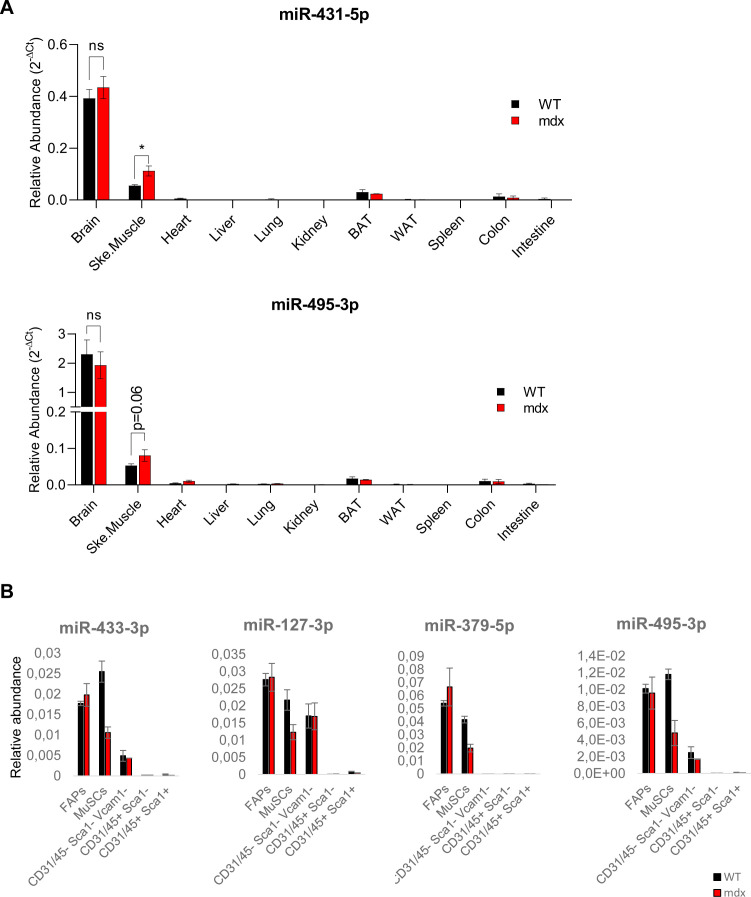
DD-miRNAs expression level in different organs, tissues and skeletal muscle cellular subpopulations. **(A)** Comparison of expression levels of 2 representatives DD-miRNAs (miR-431-5p and miR-495-3p) between different organs from 5-wk-old wild-type and mdx mice (n = 3–4). **(B)** Comparison of expression levels of four representative DD-miRNAs between different FACS-sorted cell populations from 5-wk-old mdx mice and its healthy controls (n = 3). Muscle mono-nucleated cells (MMNC) fractions were FACS-sorted by using CD31, CD45, Sca1, and Vcam1 markers from hind limb muscles of mdx and control mice. We quantified the expression of four representative DD-miRNAs (n = 3) in five subpopulations that are typically found in skeletal muscle, which are the hematopoietic cells (CD45^+^), endothelial cells (CD31^+^), fibro-adipocyte progenitors (FAPs) (CD31−/CD45−/Sca1+), satellite cells (CD31−/CD45−/Sca1−/Vcam1+), and cells that are negative for all four markers (CD45−/CD31−/Sca1−/Vcam1−). Expression of DD-miRNAs was detected in FAPs, satellite cells, and CD31/CD45/Sca1/Vcam1 negative cellular fraction. Comparison between the mdx and WT cells showed that DD-miRNAs were expressed to similar levels in the FAPs and in the negative cell population. In contrast, a lower DD-miRNAs expression was detected in satellite cells derived from the mdx mouse compared with control, which is consistent with the activated state of satellite cells of the mdx mouse, and with the fact that DD-miRNAs are known to be down-regulated in activated satellite cells (Castel et al, 2018). Data are presented as mean ± SEM. *P < 0.05; **P < 0.01; ***P < 0.001.

In addition to the muscle, the postnatal expression of DD-miRNAs was shown also in the brain (Seitz et al, 2004; Gao et al, 2015). In agreement, we found the highest expression DD-miRNAs in the brain, which was followed by the skeletal muscle, and a much lower levels in all other tissues and organs. However, the up-regulation of DD-miRNAs in muscular dystrophy was found in the mdx mouse only in the skeletal muscle (Fig S1A). Thus, serum up-regulation of DD-miRNAs in this mouse model is likely originating from the muscle. Because the skeletal muscle is a complex tissue composed of several cell types, we attempted to clarify which are the specific sites and cells that are expressing the DD-miRNAs within the muscle. We performed in situ hybridization (ISH) of two relatively highly expressed DD-miRNAs, miR-127-3p and miR-379-5p, as well as MyomiR miR-1-3p, in the diaphragm of 5-wk-old mdx mice and their respective controls. Both DD-miRNAs presented similar pattern of high expression in miR-1–positive centrally nucleated myofibers (Fig 1F), indicating that regenerating myofibers may contribute to the up-regulation of DD-miRNAs in the serum, although contribution of other cell types, tissues and organs cannot be excluded. We therefore profiled DD-miRNAs in FACS-sorted muscle mono-nucleated cell (MMNC) subpopulations, and in vitro differentiated myotubes. DD-miRNAs expression was not up-regulated in any of the mdx-derived MMNC fractions. On the contrary, in agreement with (Castel et al, 2018), DD-miRNAs expression was repressed in the freshly sorted satellite cells (QSC) (Fig S1B). To test DD-miRNAs expressions in myotubes, satellite cells from hind-limbs muscles of mdx and control mice were FACS-sorted, grown, and differentiated in vitro for 5 d. Profiling of three representative DD-miRNAs confirmed their reduced level in mdx QSC. DD-miRNAs expression then dropped down sharply at the beginning of the differentiation stage. Expression, however, raised up in day-5 differentiated myotubes, to a level significantly higher in mdx than in controls (Fig 1G, n = 3). Thus, muscle up-regulation of DD-miRNAs is seemingly coming from regenerating myofibers. In summary, the postnatal expression of DD-miRNAs in the mouse is the highest in brain, followed by skeletal muscle. In various muscular dystrophies, DD-miRNAs expression is up-regulated both in muscle and serum. In the muscle, the up-regulation is occurring in the regenerating fibers. The data could also suggest that serum up-regulation of an active mechanism rather than passive leakage through damaged sarcolemma because these miRNAs expression is up-regulated both in muscle and serum. In the muscle, the up-regulation is occurring in the regenerating fibers. DD-miRNAs is linked to their increased expression in regenerating myotubes, likely through regenerated fibers are not in the process of degeneration. The data could also suggest that serum upregulation of DD-miRNAs is linked to their increased expression in regenerating myotubes, likely through an active mechanism rather than passive leakage through damaged sarcolemma, since these regenerated fibers are not in the process of degeneration.

### Intramuscular administration of AAV-DD-miRNAs resulted in reduced muscle mass and myofiber diameter

**Figure 2.**
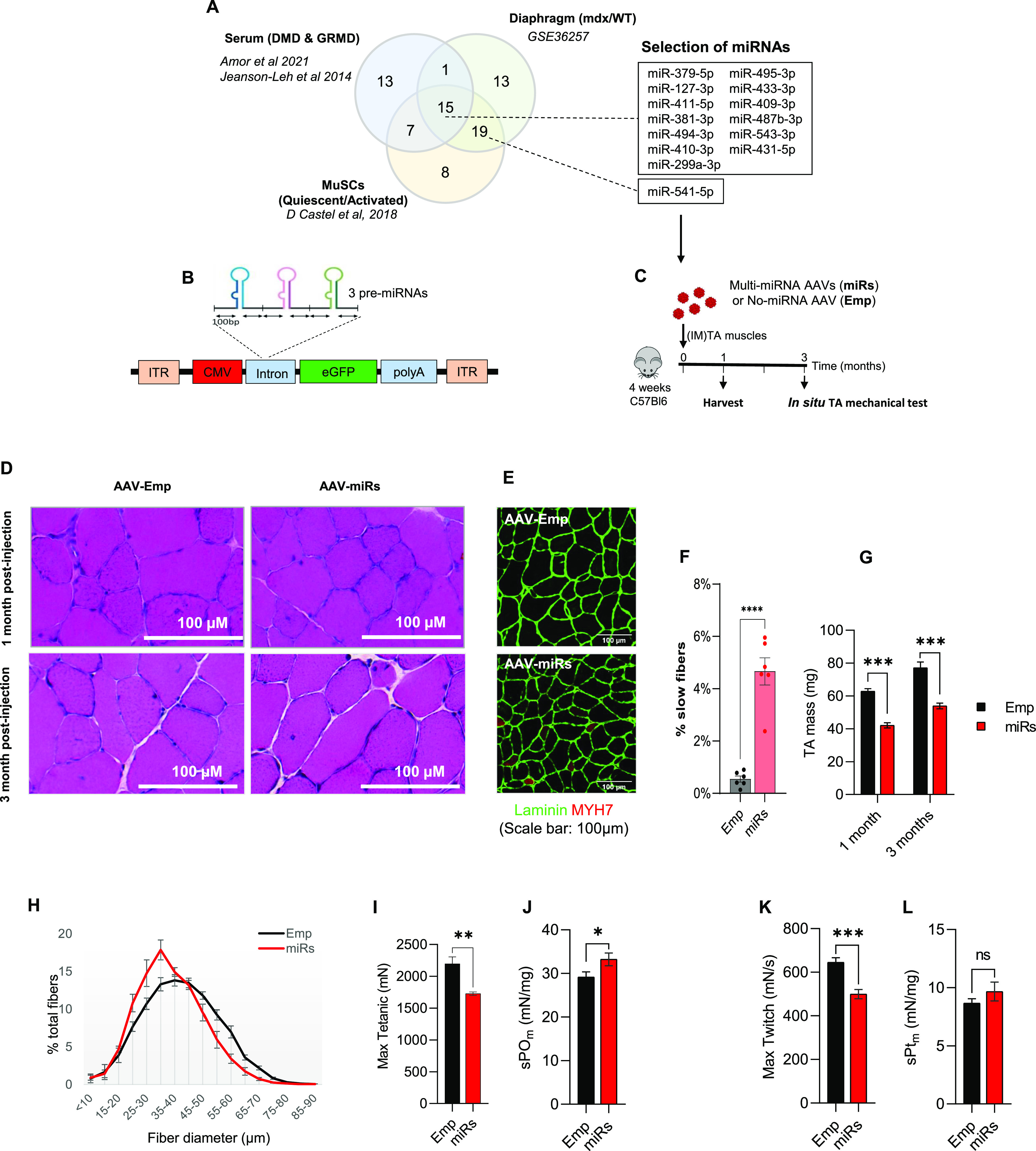
Intramuscular administration of AAV-DD-miRNAs resulted in reduced muscle mass and myofiber diameter. **(A)** Of the selected 14 DD-miRNAs, 13 are commonly dysregulated in the serum of Duchenne muscular dystrophy patients and GRMD dog models (Jeanson-Leh et al, 2014; Amor et al, 2021), in the diaphragm muscle of the mdx mouse (GEO accession: GSE3625). **(B)** AAV construction strategy for the overexpression of 14 DD-miRNAs by the co-administration of 5 AAV vectors. **(C)**. Experimental design of the in vivo overexpression of DD-miRNAs by the intramuscular co-injection of 5 AAV vectors (1 × 10^10^ VG/kg all AAV vectors together) to the TA muscle of a wild-type 4-wk-old mouse (miRs). Injection of the same titer (1 × 10^10^ VG/kg) AAV vector with the same expression cassette but pre-miRNAs sequences at the same titer (Emp) served as control in the experiment. **(D)** H&E staining of the treated TA muscles, 1 and 3 mo post injection. **(E, F)** Myosin heavy chain-7 (MYH7) staining for the of the treated TA muscles (E), and its quantification (F), 1 mo post injection. **(G)** Masses of TA muscles 1 mo and 3 mo post injection (n = 10). **(H)** TA muscle myofiber’s diameter distribution 1 mo post injection (n = 8). **(I, J, K, L)** In situ max tetanic (I) and max twitch (J) forces of treated muscles 3 mo post injection (n = 5), and its normalization to muscle masses (K, L). Data are presented as mean ± SEM. Statistics were performed with *t* test. *P < 0.05; **P < 0.01; ***P < 0.001.

**Figure S2.**
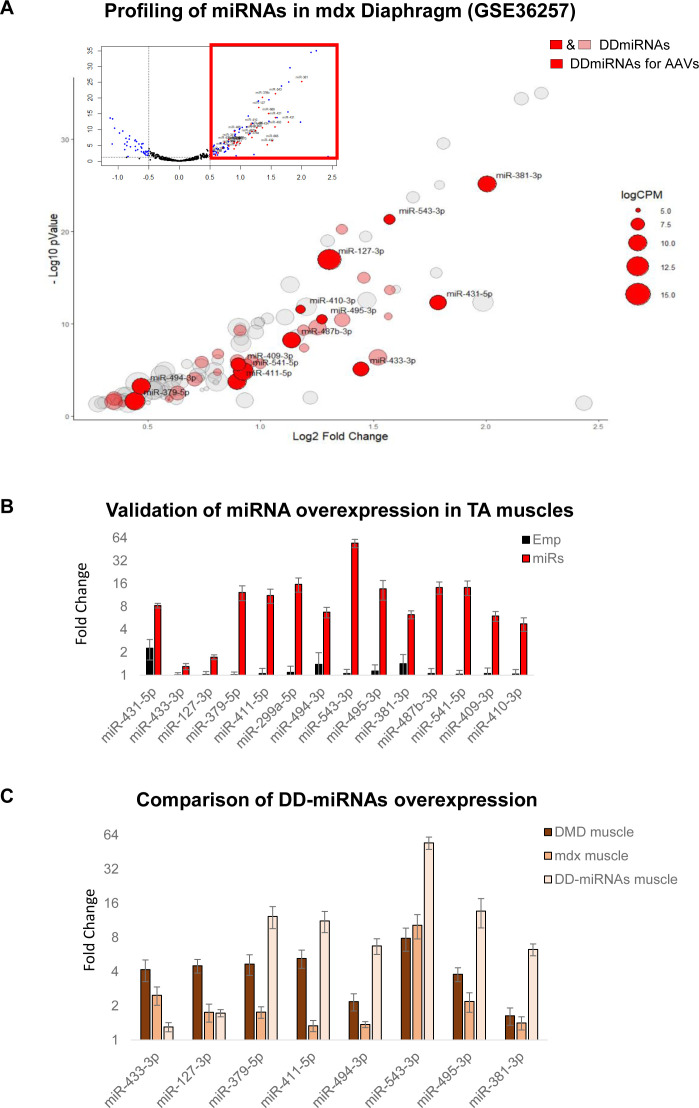
Selection and in vivo overexpression of 14 DD-miRNAs in skeletal muscle. **(A)** Volcano plot of miRNA profiling of the diaphragm of 8-wk-old mdx and control mice (upper part), and a zoom-in of significantly up-regulated miRNAs in mdx muscle (lower part) (data were taken and reanalyzed from GEO: GSE36257 [Roberts et al, 2012]). Expression levels are presented by the size of the circle. DD-miRNAs are colored (pink or red). MiRNAs in red were selected for the overexpression by AAV vectors in the present study. **(B)** Validation of DD-miRNAs overexpression in the treated mice. TA muscles were analyzed 1 mo after injection with AAV-DD-miRNAs (miRs) compared with AAV-Empty control (Emp) (n = 6). **(C)** Comparison of DD-miRNAs overexpression in different systems: muscle biopsies from Duchenne muscular dystrophy patients compared with healthy controls (Fig 1A), diaphragm muscle of mdx mice compared with C57Bl10 control (Fig 1B), and DD-miRNAs overexpression in the TA muscle of a healthy mouse by AAV vectors. Data in (B, C) are presented as mean ± SEM.

To investigate the functions of DD-miRNAs in vivo in skeletal muscle and because miRNAs often act synergistically, we therefore overexpressed simultaneously a selection of miRNAs in the wild-type muscle using recombinant adeno-associated virus (AAV-derived vectors). We selected 14 DD-miRNAs (Fig 2A), according to their level of expression and dysregulation in dystrophic serum, skeletal muscle and quiescent satellite cells (Fig S2A and Table S1).

Table S1 Selection of DD-miRNAs for overexpression in muscle.

The mouse pre-miRNA sequences of these 14 DD-miRNAs were subcloned into five AAV expression vectors (four AAV vectors with three DD-miRNAs each, and one AAV vector with two DD-miRNAs) that co-express the GFP protein, as schematically described in Fig 2B. These five AAV vectors were co-administrated by an intramuscular (1 × 10^10^ VG/TA muscle) injection into the tibialis anterior (TA) muscle of 4-wk-old C57Bl6 control mice. An AAV vector with the same expression cassette but devoid of pre-miRNA sequences was administrated (at the same final titer of 1 × 10^10^ VG/TA muscle) as negative control. The protocol included two time points (1 and 3 mo post injection) with a muscle function tests at the 3-mo group (Fig 2C). Analysis of the injected muscles confirmed the overexpression of all injected DD-miRNAs (Fig S2B) to approximately the levels of the endogenous DD-miRNAs in the diaphragms of the mdx mice and of DMD muscle biopsies (Fig S2C). Histological hematoxylin and eosin staining reveals an occasional centronucleated myofibers, which is commonly observed at the site of injection in the intramuscularly administrated AAV vector, to a similar level in all injected muscles (Fig 2D). Myosin heavy chain-7 (MYH7) staining identified increased expression of slow oxidative fibers in the DD-miRNA-overexpressed muscles, 1 mo post injection, which remained below 5% of the all myofibers (Fig 2E and F). DD-miRNAs overexpressing TA muscles presented a significantly reduced muscle mass at both 1 and 3 mo after injection (49.13% and 43.15%, respectively; Fig 2G), and smaller myofibers, demonstrated by the shift to the left of the fiber-size distribution curve (Fig 2H), 1 mo after injection. At 3 mo post injection, in situ mechanical force measurement showed that both absolute value of maximum tetanic force and maximum twitch force of DD-miRNA overexpressed muscles significantly dropped at 27.2% and 29.1%, respectively (Fig 2I and J, n = 5). When reported to the muscle mass, the normalized values of tetanic force and twitch force were slightly higher in the presence of DD-miRNAs (Fig 2K and L, n = 5), indicating that the reduction in mechanical force was due to the loss of muscle mass.

### The transcriptomic changes after ectopic overexpression of 14 DD-miRNAs overlap with the dysregulation in mdx dystrophic muscle

**Figure 3.**
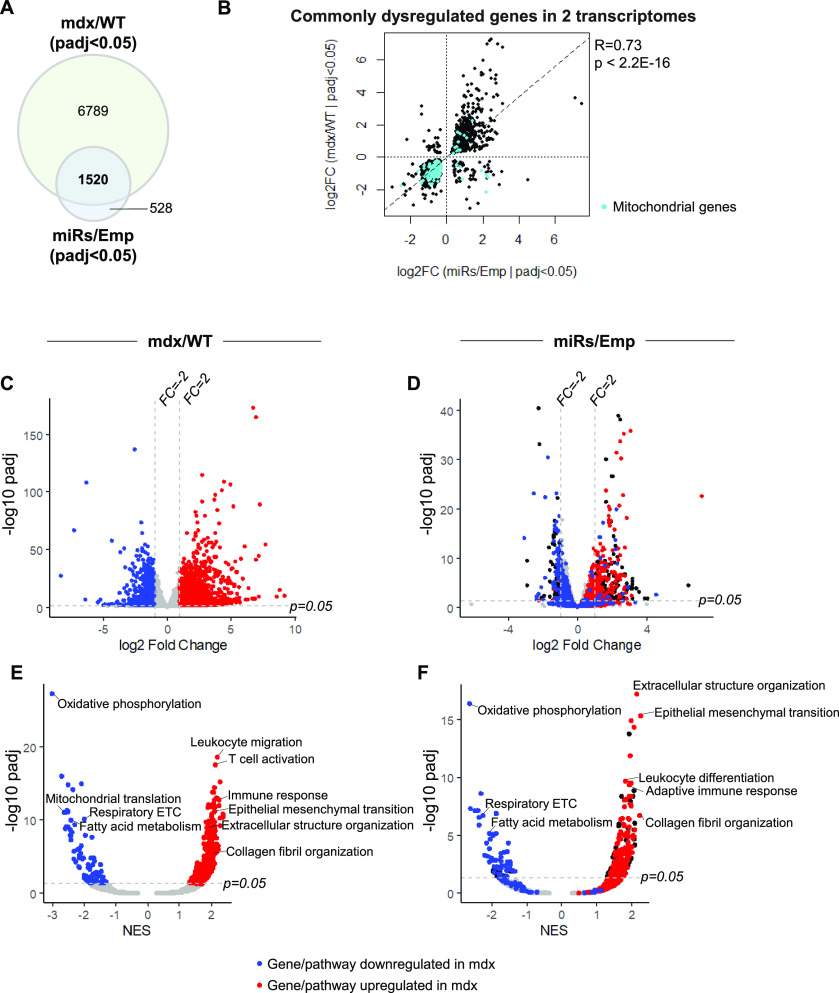
Comparison of the transcriptomic profiles between 14 DD-miRNAs ectopic expression and the mdx dystrophic muscle. **(A)** Venn diagram of dysregulated genes in mdx diaphragm compared with wild-type control and in DD-miRNA-overexpressed TA muscle compared with AAV control. **(B)** Dot plot of log_2_ fold change values of 1,520 commonly dysregulated genes in the two transcriptomes and its Pearson correlation test. Cyan dots represent mitochondrial-related genes (Mitocarta 2.0). **(C, D)** Volcano plots of mdx/WT transcriptome (C) and miRs/Emp transcriptome (D). Blue and red dots are of, respectively, down- and up-regulated (FC_mdx/WT_ > 2, adjusted *P*-value < 0.05) genes in C (mdx versus WT) The same genes and their coloration is projected on the graph in D, of (miRs/Emp). **(E, F)** Volcano plots of all Hallmark and Gene Ontology (Biological Processes) gene sets from GSEA analysis, comparison of mdx/WT transcriptome (E) and miRs/Emp transcriptome (F). Blue and red dots are of, respectively, down- and up-regulated (adjusted *P*-value < 0.05) Biological Processes in (E) (mdx versus WT). The same Biological Processes and their coloration are projected on the graph in (F), of (miRs/Emp). This visualization demonstrates high-level similarity of up and down-regulation of genes and biological processes between the two systems.

To understand to which extend the dysregulation of DD-miRNAs contributes to the transcriptomic changes of the dystrophic muscle, we compared the transcriptomic profile of the dystrophic muscles with that of the healthy muscles overexpressing DD-miRNAs. In the dystrophic diaphragm, 8,309 differentially expressed genes (DEGs) were identified (n = 4, BH-adjusted *P*-value < 0.05) (Fig 3A and C). In 1-mo-treated muscles injected with AAV-DD-miRNAs, DD-miRNAs overexpression resulted in 2048 DEGs (n = 3, BH-adjusted *P*-value < 0.05) (Fig 3A and D), of which 74.2% (1,520 of 2,048) were also dysregulated in the dystrophic muscle (Fig 3A). Furthermore, a highly significant correlation was observed between the level of dysregulation of these 1,520 common DEGs within the two datasets (Pearson correlation test, R = 0.73, *P*-value < 2.2 × 10^−16^) (Fig 3B). These data indicate that most of the changes driven by DD-miRNAs overexpression in the normal muscle are included in the transcriptomic dysregulation seen in the mdx muscle. Of note, we found that many of the highly correlating repressed transcripts are of mitochondrial genes (Cyan dots in Fig 3B).

Next, Gene Set Enrichment Analysis was used to interpret the transcriptomic changes at the pathway level (Liberzon et al, 2015). As expected, pathways related to immune response and fibrosis progression were found highly up-regulated in the mdx muscle, and many metabolic pathways were significantly down-regulated (Fig 3E). Strikingly, high-level overlap of pathway dysregulation was identified in the DD-miRNA-overexpressed muscle, compared with negative controls (Fig 3F and Table S2). Consistent with the down-regulation of mitochondrial transcripts, the pathway analysis revealed down-regulation of mitochondrial pathways and particularly of oxidative phosphorylation, which was by far the most down-regulated pathway in both systems (the dystrophic muscle and the healthy muscles overexpressing DD-miRNAs).

Table S2 Gene Set Enrichment Analysis of transcriptomes from dystrophic muscle and DD-miRNA-overexpressed muscles.

Taken together, the data supported that about 75% of the transcripts which are potentially repressed by DD-miRNAs, may participate in significant gene regulation in the dystrophic diaphragm of the mdx mouse, which potentially account to up to ∼20% of the diaphragm transcriptome dysregulation.

### Dlk1-Dio3 miRNAs affect metabolic pathways in the skeletal muscle

**Figure 4.**
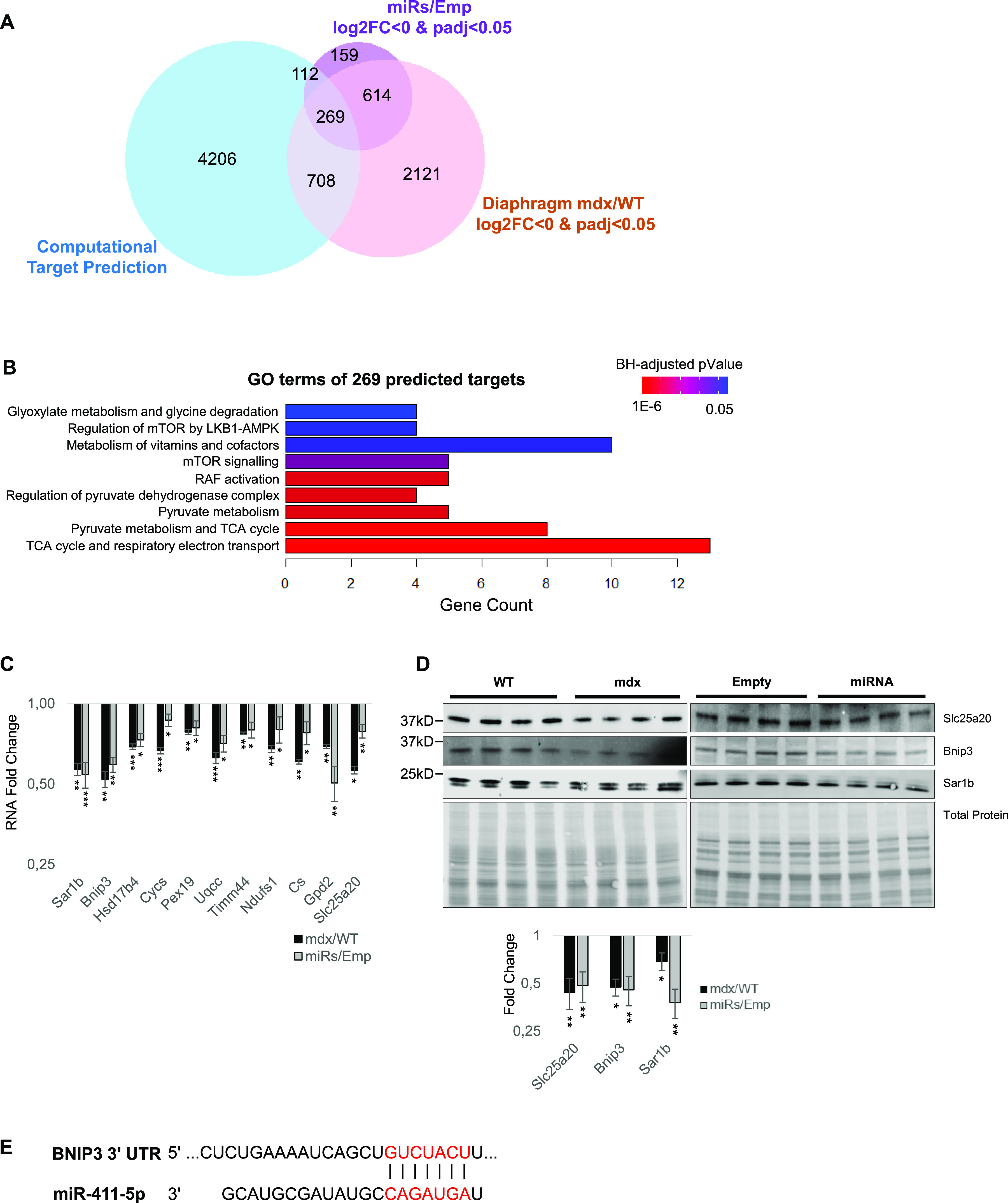
DD-miRNAs affect metabolic pathways in the skeletal muscle. **(A)** Venn diagram of significantly down-regulated genes in mdx/WT, of miRs/Emp transcriptome and of DD-miRNAs’s predicted target genes. **(A, B)** Pathway analysis of the 269 predicted targets for DD-miRNAs identified in (A). **(C, D)** Validation at RNA (C) and protein (D) levels of selected predicted targets (n = 4). **(E)** Bnip3 is a predicted target of miR-411-5p. Data in (C) and (D) are presented as mean ± SEM. Statistics were performed with *t* test. *P < 0.05; **P < 0.01; ***P < 0.001.

Next, we used a bioinformatics approach to identify potential target genes for the 14 selected DD-miRNAs. First, five prediction tools (DIANA, Targetscan, PicTar, Miranda, and miRDB) were used, generating 5,295 candidate targets predicted by at least two different tools (Fig 4A). The list of these predicted targets was subsequently crossed with the lists of genes down-regulated (log_2_FC < 0 and *P*adj < 0.05) in DD-miRNAs injected muscles and in the mdx diaphragm (that naturally overexpresses the DD-miRNAs). The analysis resulted in 269 DD-miRNAs predicted targets (Table S3 and Fig 4A). These 269 genes were subjected to gene ontology (GO) classification, which identified metabolism-related terms at the highest *P*-values, including mTOR signaling, pyruvate metabolism, TCA cycle and mitochondrial electron transport chain (Fig 4B). Down-regulation of a selection of these genes was then experimentally validated in the dystrophic muscle (5-wk-old mice, diaphragm muscle, n = 4) and AAV-DD-miRNA-injected TA muscles (n = 4) at the levels of mRNA (Fig 4C) and protein (Fig 4D). Among the tested genes we included the OxPhos complex I protein Ndufs1, the electron transport chain cytochrome C (CycS), the Oxphos complex III protein Uqcc, and the mitochondrial Bnip3. Of the strongest affected DD-miRNA targets was Bnip3, predicted to be targeted by miR-411-5p (Fig 4C–E). Of interest, Bnip3, which regulates essential mitochondrial functions including mitophagy and apoptosis, is thought to be a potential therapeutic target for diseases of secondary mitochondrial dysfunction (Gao et al, 2020), and was identified recently as down-regulated in DMD in the context of dysfunctional autophagy (Gao et al, 2020) and mitophagy (Luan et al, 2021). Taken together, these data support strongly that DD-miRNAs are involved in the metabolic adaptive response of the dystrophic muscle.

Table S3 List of bioinformatics-predicted target genes for the selected 14 DD-miRNAs.

### DD-miRNAs affect mitochondrial functions in the dystrophic muscle

**Figure S3.**
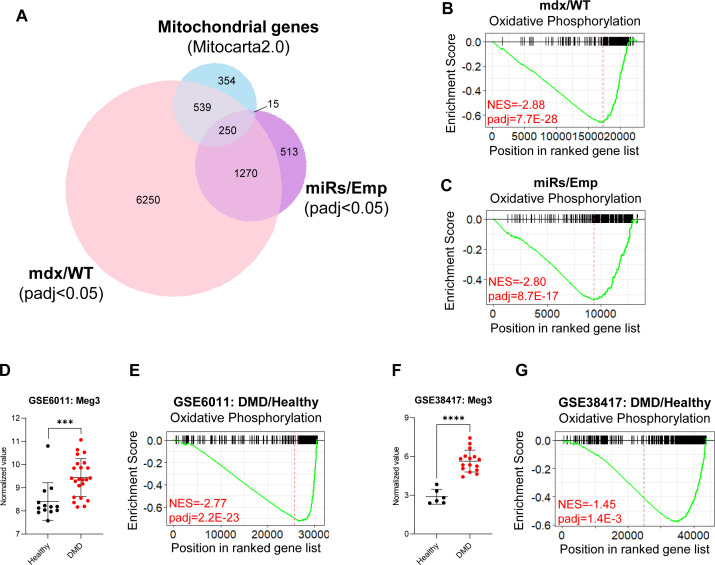
DD-miRNAs affect mitochondrial functions in the dystrophic muscle. **(A)** Venn diagram of mitochondria-related genes (Mitocarta 2.0) and of significantly dysregulated genes, in mdx/WT and miRs/Emp transcriptomes. **(B, C)** GSEA analysis of OxPhos gene set comparing mdx versus WT diaphragms (A) and miRs versus Emp TA muscles (B). **(D, E, F, G)** Expression level of Meg3 transcript and GSEA analysis of OxPhos gene set in muscle biopsies of Duchenne muscular dystrophy patients compared with healthy controls in two different datasets. Data were taken and reanalyzed from GEO GSE6011 (Pescatori et al, 2007) (D, E) and GEO GSE38417 (F, G). Data are presented as mean ± SD. *P < 0.05; **P < 0.01; ***P < 0.001.

**Figure 5.**
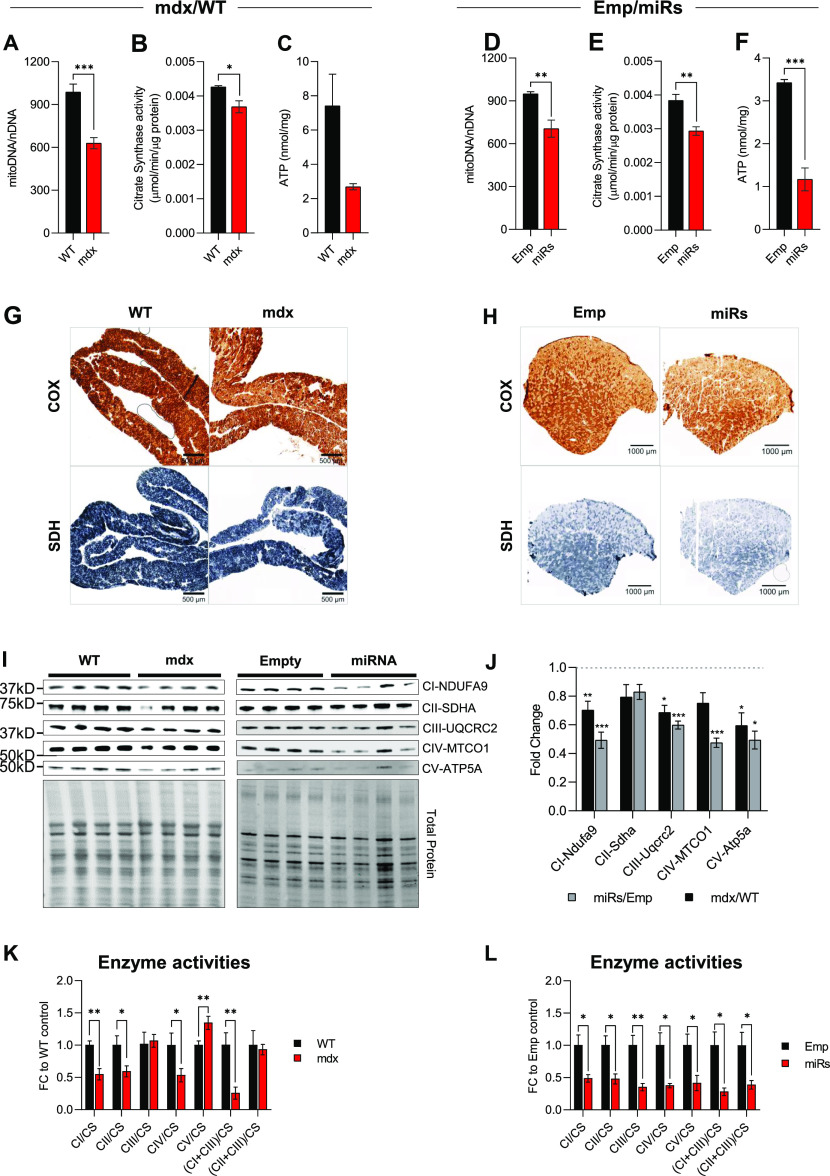
DD-miRNAs affect mitochondrial functions in the dystrophic muscle. **(A, D)** Levels of mitochondrial DNA in mdx versus WT diaphragm muscles (A, n = 6) and miRs versus Emp TA muscles (D, n = 4). **(B, E)** Citrate Synthase activities in mdx versus WT diaphragm muscles (B, n = 6) and miRs versus Emp TA muscles (E, n = 6). **(C, F)** ATP concentration in mdx versus WT diaphragm muscles (C, n = 6) and miRs versus Emp TA muscles (F, n = 6). **(G, H)** Staining of COX (upper panels) and SDH (low panels) activities in mdx versus WT diaphragm muscles (G) and miRs versus Emp TA muscles (H). **(I, J)** Western blots of five representative proteins for five OxPhos complexes comparing mdx versus WT diaphragm muscles and miRs versus Emp TA muscles (I, n = 4), and their quantification (J). **(K, L)** Activities of different OxPhos enzymes comparing mdx versus WT diaphragm muscles (K, n = 5–6) and miRs versus Emp TA muscles (L, n = 6). Data are presented as mean ± SEM. Statistics were performed with *t* test. *P < 0.05; **P < 0.01; ***P < 0.001.

Clustered miRNAs are thought to coordinately regulate genes that are functionally related (Wang et al, 2016). In the dystrophic muscle, we have recently shown that one specific DD-miRNA, namely, miR-379-5p, is acting on the mitochondrial oxidative phosphorylation pathway (Sanson et al, 2020). Taken together, we hypothesized that, in the dystrophic muscle, DD-miRNAs might coordinately regulate mitochondrial functions. To estimate the global level of mitochondrial adaptation that occurs in the dystrophic muscle, we used our transcriptomic data of the mdx diaphragm and of the DD-miRNA–overexpressed muscles, combined with the MitoCarta database of mitochondrial genes (Calvo et al, 2016). Of the 8,309 dysregulated (adjusted *P*-value < 0.05) transcripts in the mdx diaphragm, 789 (9.50%) were found to be mitochondrial. To estimate the relative role DD-miRNAs in this mitochondrial adaptive response, we found that of the 2,048 dysregulated transcripts in the TA-DD-miRNAs, 265 (12.94%) were found to be mitochondrial (Fig S3A), of which 250 (of 265) were commonly dysregulated in the mdx diaphragm and therefore potentially regulated by DD-miRNAs in the mdx diaphragm. Thus, of the 789 mitochondrial transcripts dysregulated in the mdx diaphragm, about one-third could be impacted by DD-miRNAs. This is, however, an underestimation because only 14 of all dysregulated DD-miRNAs (Jeanson-Leh et al, 2014; Amor et al, 2021), were ectopically overexpressed in the TA muscle. We then attempted to characterize in details the mitochondrial effect of the DD-miRNA–overexpressed muscle as compared to the mdx diaphragm. Mitochondrial DNA copy number (Fig 5A and D), Citrate Synthase (CS) levels (Fig 5B and E), and resting ATP concentration level (Fig 5C and F) were both significantly reduced in mdx compared with WT and in TA-DD-miRNAs compared with TA-Empty control. Immunohistochemistry of cytochrome oxidase (COX) and succinic dehydrogenase in mdx diaphragm and TA-DD-miRNAs muscle confirmed a reduction of mitochondrial activities in those two muscles (Fig 5G and H). Taken together, the data support that DD-miRNAs are acting on mitochondrial metabolism in dystrophic muscle. The gene expression and pathway analyses described above indicated that mitochondrial oxidative phosphorylation might be one of the most affected mitochondrial functions downstream to the dysregulation of DD-miRNAs (Figs 3E and F and S3B and C). We therefore attempted to characterize it further. Similar reduction of OxPhos RNA level was observed in muscle biopsies from two independent large cohorts of young DMD patients compared with its age-matched healthy controls (Fig S3E and G) (data were taken from GEO accession: GSE6011 [Pescatori et al, 2007] and GEO accession: GSE38417). As expected, expression level of Meg3 transcripts are highly significantly up-regulated within the DMD groups’ muscles (GSE6011: n = 13/23, *P*-value = 0.0009; GSE38417: n = 6/16, *P*-value < 0.0001) (Fig S3D and F). These data demonstrate a reduced OxPhos and its negative correlation with Dlk1-Dio3 maternal transcripts in muscle biopsies of human DMD patients too.

A Western blot analysis demonstrated reduced expression of proteins belonging to all four mitochondrial respiratory chain complexes and ATP synthase in the both mdx versus control diaphragms and DD-miRNAs versus control transduced TA muscle (Fig 5I and J). Of interest, of the five proteins that were monitored in this experiment, two are potential targets of DD-miRNAs (SDHA is a potential target of miR-411-5p, and UQCRC2 is of miR-409-3p), whereas the other three (CI-NDUFA9, CIV-MTCO1, and CV-ATPA5) are not predicted to be direct targets of any DD-miRNA. It indicates that the down-regulation of OxPhos proteins in the conditions of elevated DD-miRNA in the muscle involved a global down-regulation of mitochondrial complexes, rather than (or in addition to) direct targeting by DD-miRNAs. We then tested the activities of the different mitochondrial complexes (normalized to CS activity – an indicator of mitochondrial mass) (Spinazzi et al, 2012). Reduced complex activities were observed in complexes I, II, and IV in both systems, whereas complex III and V presented reduced activity only in the TA-DD-miRNAs muscles (Fig 5K and L). Significant reductions were also detected for the combined activity of complex I + III (NADH cytochrome c oxidoreductase) in both systems.

**Figure 6.**
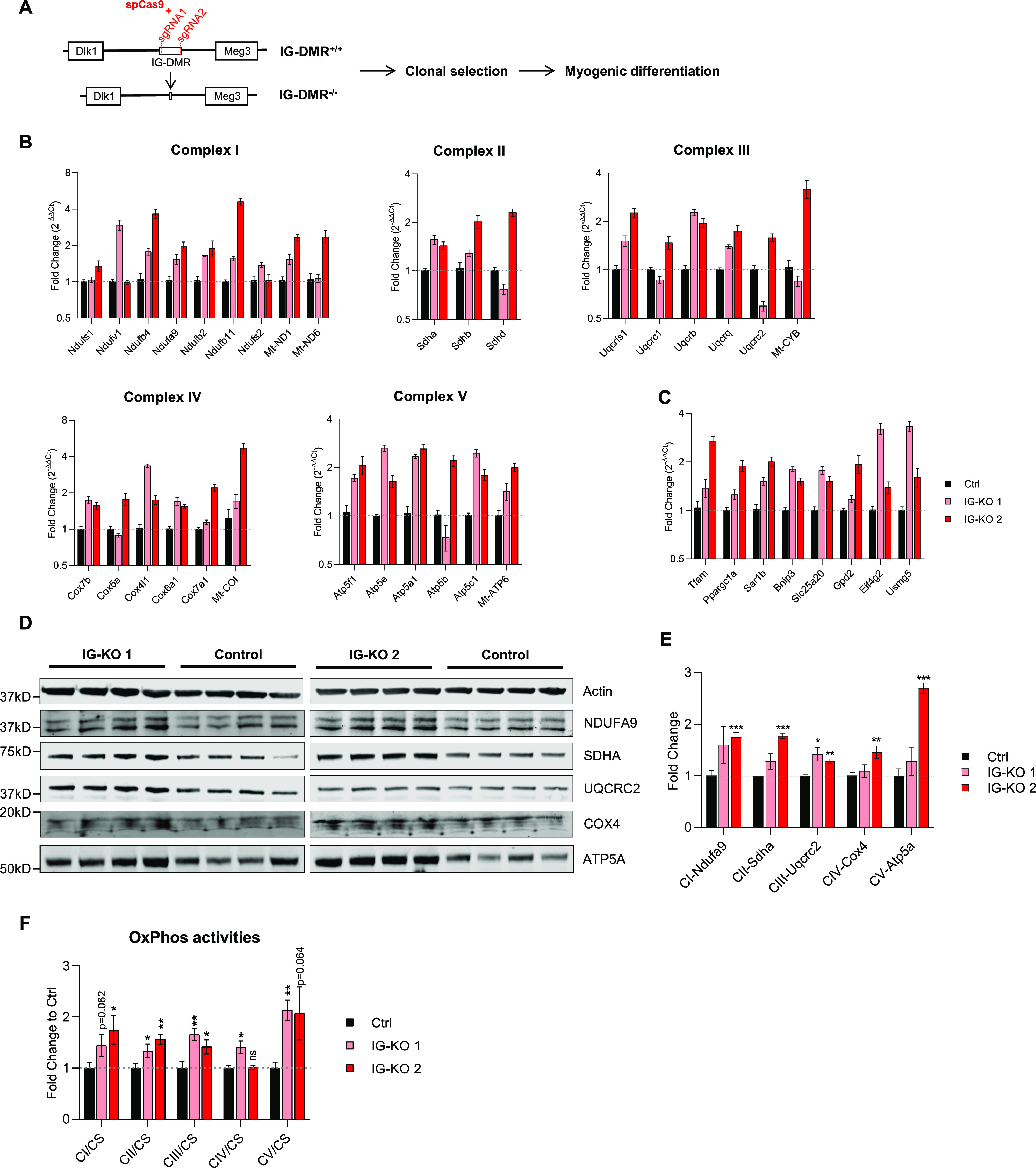
Knocking down DD-miRNAs results in increased OxPhos expression and activity. **(A)** Diagram of IG-DMR deletion by CRISPR-Cas9. **(B, C, D, E, F)** Analysis of DD-miRNAs knocked-down iPS clones compared with control (2 iPS clones, n = 4). (B), RNA level of selected transcripts of five OxPhos complexes and (C), predicted DD-miRNA targets; (D), Western blot of five representative proteins for five OxPhos complexes, and (E), its quantification. (F), CS-normalized activity of five OxPhos complexes. Data presented as mean ± SEM. Statistics were performed with *t* test. *P < 0.05; **P < 0.01; ***P < 0.001.

**Figure S4.**
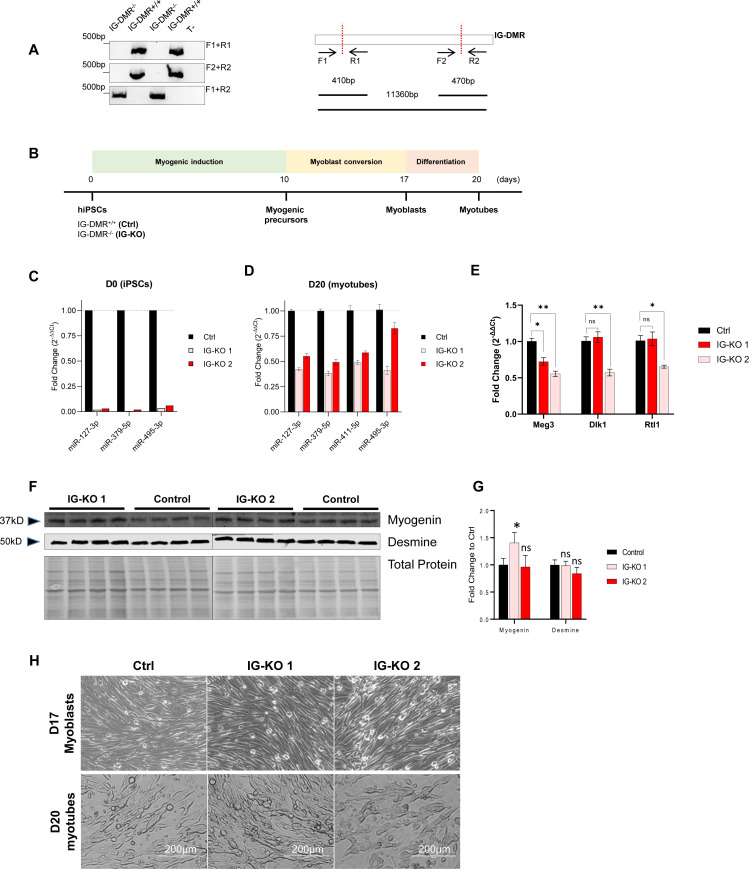
Biallelic deletion of IG-DMR in hiPSCs. **(A)** Confirmation of 11-kb IG-DMR deletion in different IG-DMR^−/−^ clones by PCR using different sets of primers (left). Positions of primers used in PCR are illustrated (right). **(B)** Diagram of the myogenic differentiation of hiPSCs. **(C, D)** Relative expression levels of representative DD-miRNAs at D0 (C - hiPSCs) and D20 (D - myotubes) in 2 IG-DMR^−/−^ clones compared with control (n = 4). Data are presented as mean ± SEM. **(E)** Expression of non-miRNA transcripts (Meg3, Dlk1, and Rtl1) of the DLK1-DIO3 locus is slight reduced in the IG-KO lines. **(F, G, H)** Western analysis (F), and its quantification (G), of the muscle differentiation markers myogenin and desmin. **(H)** Representative images of D17 myoblasts (upper panels) and D20 myotubes (lower panels) of different IG-DMR^−/−^ and control clones.

Next, we wanted to ask whether mitochondrial activities could be enhanced by reducing DD-miRNAs expression. Because no viable mouse model exists with a silencing of the entire DD-miRNA cluster, we decided to create in vitro myogenic model of reduced DD-miRNAs expression. The M180 human induced pluripotent stem cell (hiPSC) line was selected because it expresses high-level DD-miRNAs throughout in vitro myogenesis (Massouridès et al, 2015). Because it was previously shown that maternal Dlk1-Dio3 expression, including DD-miRNAs, was abolished in IG-DMR^−/−^ mouse embryonic stem cells (Das et al, 2015) we deleted, in M180 iPS, the 11-kb IG-DMR by double-cut CRISPR/spCas9 strategy, with two sgRNAs target the two ends of IG-DMR (Fig 6A). Clonal selection was performed and biallelic deletion verified (Fig S4A). IG-DMR^+/+^ (Ctrl) and IG-DMR^−/−^ (IG-KO) clones were differentiated in vitro into skeletal muscle myotubes (Fig S4B–H). Whereas complete KO of DD-miRNA was seen in the iPS pluripotent state (D0) (Fig S4C), only partial, but significant, reduction was observed in fully differentiated myotubes (Fig S4D), suggesting that whereas the DD-miRNA expression is controlled solely by IG-DMR initially, additional elements control their expression after maturation. Not unexpectedly, the analysis of non-miRNA transcripts of the DLK1-DIO3 locus identified slight reduction of their expression in the IG-KO lines (Fig S4E), supporting that downstream effects may be mediated not exclusively by the targets genes of DD-miRNAs. IG-KO clones showed normal expression levels of the myogenic differentiation markers myogenin and desmin (myogenein but not desmin slightly elevated in one of the IG-KO clones) (Fig S4F and G), and correctly differentiated into myotubes (Fig S4H). We then investigated the mitochondrial responses in hiPSC-derived myotubes with reduced DD-miRNA levels. At the RNA level, IG-KO clones showed a global up-regulation of transcripts of all five OxPhos complexes, although with variation between clones in the level of some genes (n = 4, *t* test) (Fig 6B). Importantly, increased mRNA levels, in both IG-KO clones, were detected in six predicted DD-miRNAs target genes, including two master regulators of mitochondrial biogenesis (Tfam and Ppargc1a), and two validated targets (Eif4g2, Usmg5) (Sanson et al, 2020) (Fig 6C). Similarly, the protein levels of five representatives for five OxPhos complexes in the two IG-KO clones showed a global increase compared with the control (Fig 6D and E). More importantly, activities of all five OxPhos complexes in the KO clones increased significantly compared with control (Fig 6F). Accumulatively, these results support that reduced DD-miRNAs can enhance mitochondrial activity, particularly the OxPhos system in differentiated myotubes.

## Discussion

In previous investigations, we identified the up-regulation of DD-miRNAs in the serum and the muscles of DMD patients and animal models (Jeanson-Leh et al, 2014; Sanson et al, 2020; Amor et al, 2021). In the present report, we are bringing new evidences supporting a role for the DD-miRNA cluster in the regulation of mitochondrial functions in DMD.

### Indications that circulating DD-miRNAs are produced and secreted by the regenerating muscle

In agreement with previous studies (Seitz et al, 2004; Gao et al, 2015), we detected in adult mouse the highest DD-miRNAs expression in the brain, followed by the skeletal muscle. However, activation of expression in the mdx was detected only in the muscle. In addition, induced DD-miRNAs expression was observed in the serum of other mouse models for muscular dystrophy, without brain phenotype, making it unlikely that brain DD-miRNAs contributing significantly to the up-regulation of circulating DD-miRNAs in muscular dystrophy. These data support strongly that regenerating myofibers are mostly responsible for the elevated levels of muscle and circulating DD-miRNAs in muscular dystrophy. The serum profiles of DD-miRNAs over different ages in GRMD dogs (Jeanson-Leh et al, 2014) and DMD patients (Amor et al, 2021) revealed a similar expression pattern to the mCK, of reduced dysregulation level beyond the age of 1 yr in GRMD (Jeanson-Leh et al, 2014) and of 12 yr in DMD (Amor et al, 2021). However, whereas the dropdown with age in DMD of serum mCK is thought to reflect reduced muscle mass and reduced myofiber degeneration, on the contrary, the dropdown with age of circulating DD-miRNAs is seem to best explained by reduced myofiber regeneration rate, which is consistent with the well-known exhaustion of the muscle regenerative capacity in DMD with evolution of the disease.

### The DD-miRNA cluster regulates mitochondrial OxPhos in the dystrophic muscle

The regulation of mitochondrial functions by miRNAs from the Dlk1-Dio3 locus has been reported before. In the hematopoietic system, inhibiting the AKT/mTOR signaling to modulate mitochondrial OxPhos by DD-miRNAs was shown to preserve the long-term repopulation capacity of stem cells (Qian et al, 2016). In the muscular system, the metabolic maturation of muscle precursor cells was shown to be dependent on the inhibition of the Dlk1-Dio3 miRNA cluster, which resulted in the activation of mitochondrial functions and myogenic differentiation (Wüst et al, 2018). In the context of muscular dystrophy, we recently demonstrated the participation of the Dlk1-Dio3 miR-379 in the modulation of mitochondrial activity in the dystrophic muscle (Sanson et al, 2020). However, despite this strong hypothesis concerning mitochondrial functions (Vu Hong et al, 2021), rather than a hypothesis-driven approach, we undertook a strategy of a non-supervised investigation, consisting of the in vivo co-overexpression of a 14 DD-miRNAs, and global transcriptomic bioinformatics analysis. This analysis pointed toward lipid metabolism and mitochondrial functions, in particular OxPhos, as the most dysregulated pathways downstream to the DD-miRNAs, supporting experimentally strongly that the mitochondrial OxPhos system is indeed the most affected pathway in the dystrophic muscle by the cooperative activity of the DD-miRNAs. Because the activation of DD-miRNAs was also observed in the muscle and serum of three LGMD mouse models and during regeneration of normal muscle after Notexin-induced injury, the involvement of DD-miRNAs in mitochondrial functions might be associated with diverse situations of muscle injury and regeneration. Of note, other noncoding RNA species of the Dlk1-Dio3 locus (in addition to the DD-miRNAs) may participate in the regenerative process in the dystrophic muscle. In particular, the MEG3 transcript, which is up-regulated in DMD (Fig S3D and F), was shown recently to modulate epithelial to mesenchymal transition and the TGF-β pathway in the regenerating muscle (Dill et al, 2021).

### In vivo validation of control of mitochondrial OxPhos by DD-miRNAs

We wondered to what degree the dystrophic changes in the mdx muscle might be explained by the up-regulation of DD-miRNAs. To answer this question, we overexpressed 14 DD-miRNAs in vivo in the muscle and compared transcriptomic, protein level, and mitochondrial activity of the mdx muscle to the DD-miRNA overexpressing C57Bl/6 muscle. We detected a high overlap between omics changes in the normal muscle overexpressing DD-miRNAs, and in the dystrophic muscle, naturally having elevated DD-miRNA levels. Of particular interest, the mitochondrial OxPhos, which is the highest bioinformatics-predicted target pathway for DD-miRNAs, was experimentally detected as the most down-regulated pathway in both conditions. This observation strongly supports that the DD-miRNAs are direct repressive mediators of OxPhos system in the dystrophic muscle. The up-regulated DD-miRNAs are expected to down-regulate target genes in the dystrophic muscle. As expected, therefore, we found a high-level overlap in the down-regulated transcripts (which are the direct targets of DD-miRNAs), in the normal muscle overexpressing DD-miRNAs, and in the dystrophic muscle. Surprisingly, a high overlap was found also in the up-regulated transcripts, supporting that non-direct effects of DD-miRNAs may similarly occurs in the two systems. Taken together, the data support that DD-miRNAs up-regulation plays important role in mediating omics changes and mitochondrial activity in the dystrophic muscle. The direct evaluation of the silencing of all DD-miRNAs in the mdx mouse was not possible because the knocking out of the entire DD-miRNA cluster is embryonically lethal. We selected therefore an in vitro iPS-based strategy for the modeling of DD-miRNAs silencing in the muscle. The CRISPR-Cas9 induced deletion of the IG-DMR, led to a drastically reduced DD-miRNA expression in the in vitro differentiated skeletal myotubes derived from a human iPS cell line. The analysis of mitochondrial gene expression and activity in myotubes derived from two independent clones confirmed that DD-miRNA might control mitochondrial OxPhos in the muscular system. Thus, the present study linked DD-miRNAs with the control of mitochondrial activity in the regenerating myofiber.

DD-miRNAs are highly expressed in the quiescence muscle stem cell and down-regulated upon its activation (Castel et al, 2018) or in dystrophic satellite cells (Fig 1E); however, the biological function of DD-miRNAs in muscle stem cells is yet unknown. Of note, it has recently been suggested that activation of the satellite cells in the exercised muscle is OxPhos–dependent (Abreu & Kowaltowski, 2020). It is tempting to speculate that by OxPhos inhibition, DD-miRNAs are involved in the maintenance of stem cells quiescence in the resting muscle, whereas DD-miRNA down-regulation is required for the activation of stem cells in the exercising or regenerating muscle.

### Updated model for mitochondrial dysfunction in DMD

It has been suggested long ago that the entry of Ca^2+^, which is provoked in muscular dystrophy by sarcolemma instability, is followed by a pathological cascade that includes Ca^2+^ impact on mitochondria, leading to myogenic degeneration (Wrogemann & Pena, 1976). This proposition for mitochondrial dysfunction in muscular dystrophy is still widely accepted today (Zulian et al, 2016; Mareedu et al, 2021). We have recently demonstrated that increased miR-379 expression in the dystrophic muscle interferes with the OxPhos system and ATP production. Based on this discovery, we proposed a modified model for mitochondrial dysfunction in DMD. Our updated model for mitochondrial participation in the dystrophic cascade supports that in muscular dystrophy, not only miR-379 but also many other DD-miRNAs are coordinately up-regulated and cooperatively affect mitochondrial functions in the dystrophic muscle (Vu Hong et al, 2021).

### Study limitations

The knocking down of DD-miRNA expression was committed in the present study in the M180 iPS, of a non-dystrophic origin. Ideally, the demonstration of mitochondrial response for the knocking down of DD-miRNA would had been done in a dystrophic background. Yet, M180 iPS cells were selected because of seven iPS lines that included four DMD and three controls, only M180 expressed significant level of DD-miRNAs, which allows the demonstration of a physiological effect of its knocking down. Future investigation shall link DD-miRNA down-regulation to mitochondrial response in a DMD model. Another limitation of the present study is the incomplete characterization of molecular events that link the up-regulation of DD-miRNAs to the mitochondrial response. Indeed, it cannot be excluded that altered expression of protein-coding RNAs and of lncRNAs of the DLK1-DIO3 locus contribute to the observed mitochondrial response. A more detailed molecular characterization that links DD-miRNAs dysregulation to mitochondrial response shall be addressed in future investigations. In summary, the present investigation exposes a fundamental mechanism of mitochondrial adaptation in the dystrophic muscle and therefore opens a new perspective for therapeutic intervention.

## Materials and Methods

### Animal care and use

All animals were handled according to French and European guidelines for human care and the use of experimental animals. All procedures on animals were approved by Genethon’s ethics committee under the numbers CE10-122, CE10-123, CE10-124, CE10-127, and CE12-039. C57Bl10, C57Bl6, and C57BL/10ScSn-Dmd^mdx^/J mice were obtained from Charles River laboratories. Mice were housed in a SPF barrier facility with 12-h light, 12-h dark cycles, and were provided with food and water ad libitum. Only male mice were used in the present study. The animals were anesthetized with a mix of ketamine (100 mg/kg) and xylazine (10 mg/kg), or with isoflurane, for blood samples. For intramuscular injections, a volume of 25 *µ*l containing 1.0E10vg AAV vectors was injected into each TA muscle. For the duration of the study, all animals were observed at least once a day. All animals were weighed on the day of treatment as well as on the day of the necropsy.

### Muscle biopsies

Human skeletal muscle tissues were obtained from Myobank, the tissue bank of the Association Francaise contre les Myopathies (AFM; https://www.institut-myologie.org/en/recherche-2/myobank-afm/). Open skeletal muscle biopsies were performed after informed consent, according to the Declaration of Helsinki. Muscle biopsies included in this study were derived from paravertebral (two controls and two DMD), gluteus, and tensor fasciae latae (one each control and one DMD) muscles. Details of patients and the biopsies are presented in Table 1.

**Table 1.** Human muscle biopsies.

Group	Age (yr)	Muscle	Duchenne muscular dystrophy mutation
Duchenne muscular dystrophy	8	Tensor fasciae latae	Non specify
	11	Gluteus	Deletion Exon 3-37
	15	Paravertebral	Deletion Exon 10-11
	15	Paravertebral	Deletion Exon 50-52
Healthy	14	Paravertebral	None
	14	Paravertebral	
	15	Gluteus	
	19	Tensor fasciae latae	

### AAV construction and production

The 14 pre-miRNA sequences were obtained from UCSC website (https://genome-euro.ucsc.edu), spanning 100 nucleotides before and after the mature miRNA sequences. The selected pre-miRNA sequences were then arranged consecutively which respect the genomic sequences of the miRNAs. Two or three pre-miRNAs were used per AAV construct. The constructed sequences were then synthesized (Genewiz) and subcloned into a donor plasmid (AAV-CMV-eGFP) by classical molecular biology technique. AAV9 viral vectors production was performed as already described (Lostal et al, 2014). Titration was performed by RT-qPCR using primers corresponding to the sequences of AAV ITR or eGFP. Sequences of PCR primers can be found in Table S4.

Table S4 List of primers, antibodies, reagents, R packages, and public databases used in the present study.

### Satellite cell isolation from skeletal muscles and their in vitro differentiation

Satellite cells were isolated from limb muscles as described (Liu et al, 2015), with some small modifications. All hind limb muscles of 5-wk-old mice were collected and minced by using scissors. The samples were then put in the digestion medium of DMEM with 3 U/ml Dispase II (Thermo Fisher Scientific), 0.5 U/ml Collagenase A (Sigma-Aldrich), 0.2% BSA (Sigma-Aldrich), and 1X Pen-Strep (Thermo Fisher Scientific) for 2 h at 37°C with gentle shaking. The lysates were then passed through successive strainers (100, 70, and 40 *µ*m) to eliminate fibers and debris. Subsequently, red blood cells were removed from the samples by Versalyse (Reference A09777; Beckman Coulter). The MMNCs were then blocked by Mouse BD Fc Block (Reference 553142; BD Pharmingen), stained with CD31-APC, CD45-APC, Sca1-PE, Vcam1-PE.Cy7, and viability marker 7AAD. The corresponding isotype controls were used in parallel to determine the non-specific binding of antibodies. Details of antibody panel used in FACS sorting are presented in Table S4. The cells were then FACS-sorted by Astrios Cell Sorter (Beckman Coulter). Satellite cells, marked as CD31^−^ CD45^−^ Sca1^−^ Vcam1^+^, were directly placed in the proliferating medium containing Ham’s F-10 (Hyclone) supplemented with 10% horse serum and 2.5 ng/ml bFGF (Reference 100-18B; PeproTech), and incubated at 37°C, 5% CO_2_ for 3 d. Proliferated cells were then switched into a differentiation medium containing DMEM and 5% Horse Serum (Gibco) and kept at 37°C, 5% CO_2_ for 5 d.

### RNA expression analysis

Total RNA was extracted from frozen muscles or cells using Trizol (Thermo Fisher Scientific). DNA contamination from RNA samples was subsequently removed by TURBO DNA-free kit (Thermo Fisher Scientific). For the measurement of *miRNA expression*, 500 ng of total RNA were reverse-transcribed using TaqMan MicroRNA Reverse Transcription Kit (Thermo Fisher Scientific). Quantitative PCR was performed using LightCycler 480 system (Roche), with the Applied Biosystems TaqMan MicroRNA Assays (Thermo Fisher Scientific), according to the manufacturer’s protocol. Each PCR reaction was performed in duplicate. Results obtained with the average of miR-93-5p and U6 small nuclear RNA for the normalization of the data across all samples. For the measurement of *gene expression*, 1,000 ng of total RNA was reverse-transcribed using a mixture of random oligonucleotides and oligo-dT and RevertAid H Minus First Strand cDNA Synthesis Kit (Thermo Fisher Scientific). Quantitative PCR was performed using LightCycler 480 system (Roche) with the SYBR Green PCR assays (Thermo Fisher Scientific) or Taqman Gene Expression Assay (Thermo Fisher Scientific). Each PCR reaction was performed in duplicate. Results obtained with Rplp0 (for muscle samples) or Acta1 (for in vitro cells) were used to normalize the data across samples. Relative expression fold change was calculated using 2^−∆∆CT^ method, as previously described (Livak & Schmittgen, 2001). Primers used in the study are presented in Table S4.

### RNA sequencing and transcriptomic analysis

RNA quality was first examined by Bioanalyzer 2100 (Agilent). Samples with RNA integrity number greater than 7.5 were then subjected to RNA sequencing (Genewiz). The sequencing libraries were prepared using the Stranded Total RNA Library Prep Kit (Illumina) and sequenced according to the Illumina protocol by NovaSeq instrument (Illumina), resulting in ∼20 M paired-end reads per library. Filtration and quality control was done by fastp (Chen et al, 2018). The pair-end reads were subsequently mapped into GRCm38/mm10 genome by HISAT2 (Kim et al, 2015), and count tables were produced by FeatureCount (Liao et al, 2014). DEGs were identified by DESeq2 R package (Love et al, 2014). Pathway analysis was performed in R-Studio (version 4.0.3), either by over-representation methods using Gene Ontology and ReactomePA or functional class scoring using Gene Set Enrichment Analysis. The list of R packages used in the analysis can be found in Table S4.

### Cell culture and in vitro study

hiPSCs were maintained in mTeSR medium (Reference 05850; Stemcell Technologies) on Matrigel-coated plate (Reference 356230; Corning). To differentiate hiPSCs into skeletal muscle linage, 2D directed differentiation protocol was used as previously described (Caron et al, 2016). The hiPSCs were placed in three consecutive defined media as followed: SKM01 (AMSbio) from day 0 to 10, SKM02 (AMSbio) from day 10 to 17, and SKM03 (AMSbio) from day 17 to 20 (Fig S4B).

### Genomic deletion using the CRISPR/Cas9 and screening for biallelic deletion clones

Single-guide RNAs (sgRNAs) were designed by GPP sgRNA Designer (Doench et al, 2016) and synthetic oligos with chemical modifications (2′-O-Methyl at three first and last bases, 3′ phosphorothioate bonds between first three and last two bases) were synthesized by Synthego. Different combinations of sgRNAs targeting two ends of IG-DMR region were tested in 911 human cells to select sgRNAs with the highest cutting efficacy. Selected sgRNAs were then co-transfected with PX458 plasmid (#48138; Addgene), which contains the expression cassettes of *Streptococcus pyogenes* Cas9 and GFP, into hiPSCs by Lipofectamine 3000 (Thermo Fisher Scientific) according to manufacturer instruction. After 24 h post-transfection, single GFP+ cells were sorted by Astrios Cell Sorter (Beckman Coulter) directly in pre-warmed mTeSR medium supplemented with 10% CloneR (Reference 5888; Stemcell Technologies) and hES Cell Cloning & Recovery Supplement (Reference 01-0014-500; Stemgent, dilution 1/2,000). The single clones were kept at 37°C for 7 d, and the medium was changed every 2–3 d. Subsequently, genomic DNA of hiPSC clones was extracted by QuickExtract DNA Extraction Solution (Epicentre) and subjected to three PCR reactions with three sets of primers to screen for biallelic IG-DMR deletion (IG-DMR^−/−^). The positions of primers and the sizes of the PCR amplicons were illustrated in Fig S4A. The sequences of selected sgRNAs and PCR primers for screening were detailed in Tables S4 and S5. We selected two single clones with no IG-DMR deletion in both alleles from the same screening process, used as control (IG-DMR^+/+^) in subsequent experiments. Off-targets of 2 sgRNAs used in this study were identified by CHOPCHOP (Labun et al, 2019) and detailed in Tables S5 and S6. The absence of any off-target in all clones used in this study was validated by Sanger sequencing (Genewiz).

Table S5 List of sgRNAs used in the present study.

Table S6 List of off-targets associated with sgRNAs used in this study.

### Immunohistochemistry staining

The transversal sections of 8-*µ*m thickness were prepared from frozen muscles in the cryostat (Leica). For enzyme histochemistry staining of succinate dehydrogenase and Cytochrome C oxidase (COX) activities, freshly prepared slides were used and stained by using commercial kits (Reference 30114LY and 30115LY, respectively; Bio-Optica) according to the manufacturer’s protocols. Images were acquired with confocal microscopy TCS-SP8 (Leica) or AxioScan Slide Scanner (Zeiss).

### ISH of miRNAs in skeletal muscle

For miRNA ISH, muscles were dissected and immediately fixed in 10% neutral-buffed formalin overnight before embedded in paraffin blocks. Transversal sections of 4-*µ*m thickness were obtained in the Microtome (Leica). The ISH of miR-127-3p and miR-379-5p were performed by miRCURY LNA miRNA ISH Optimization Kit (FFPE) 1 (Reference 339451; QIAGEN), according to the manufacturer’s protocol. All miRNA probes are double-digoxigenin–labeled and hybridized at a final concentration of 40 nM at 53°C for 1 h. Probe with scramble sequence was used as the negative control. Nuclei were counterstained with Nuclear Fast Red (Reference 60700; Fluka). The miRNA probe sequences are shown in Table S4.

### Western blotting

Proteins were extracted from tissues or cells by RIPA buffer (Thermo Fisher Scientific) supplemented with Protease Inhibitor Cocktails (Complete PIC; Roche) and Benzonase 1:1,000 (Millipore). Total protein concentration was measured by using Pierce BCA Protein Assay (Thermo Fisher Scientific). Equal amounts of protein were then loaded and separated by precast 4–12% Bis-Tris polyacrylamide gel (Thermo Fisher Scientific). Subsequently, the protein was transferred to a nitrocellulose membrane with the iBlot2 Dry Blotting system (Thermo Fisher Scientific). For detecting the proteins of interest, the membrane was blocked in Odyssey Blocking Buffer (LI-COR) for 1 h at room temperature before incubated with primary antibodies diluted in 50% Odyssey Blocking Buffer overnight at 4°C. After washed in 1× PBST, the membrane was incubated with secondary antibodies 1/5,000 diluted in the 50% Odyssey Blocking Buffer for 1 h at room temperature. The membrane was then rewashed and blotting signals were acquired in the Odyssey Infrared Imaging system. Total protein (for muscle samples) measured by Revert 700 Total Protein (LI-COR) (for muscle samples) or Actin (for in vitro cells) was used as loading controls for quantification. Details of antibodies used in this study are presented in Table S4.

### Measurement of CS activities

CS activity of muscle extracts was determined by CS Assay Kit (Reference CS0720; Sigma-Aldrich) according to the manufacturer’s protocol. 5–10 mg of muscle tissues were initially used and 5 *µ*g of protein extract was used to measure CS activity.

### Measurement of ATP concentration

ATP concentration of the muscle biopsies was determined by ATP Assay Kit (Reference ab83355; Abcam) according to the manufacturer’s protocol. 5–10 mg of muscle tissues were used in the assay. The precise mass of muscle tissues was noted and subsequently used to normalize across samples.

### Measurement of mitochondrial and viral DNA

Total DNA was extracted from 5–10 mg of muscle tissues by using QIAamp DNA Mini Kit (Reference 51304; QIAGEN) according to the manufacturer’s protocol. Purified DNA was subjected to qPCR using SYBR Green PCR assays (Thermo Fisher Scientific). Levels of genomic DNA were used to normalize data across samples. Primers amplifying genomic DNA, mitochondrial DNA, and DNA from AAV vectors are detailed in Table S4.

### Measurement of mitochondrial OxPhos enzymatic activities

Enzymatic activities of complex I (NADH:ubiquinone oxidoreductase, rotenone-sensitive activity), II (succinate dehydrogenase, malonate-sensitive activity), III (decylubiquinol cytochrome c oxidoreductase, antimycin A - sensitive activity), and IV (cytochrome c oxidase, KCN-sensitive activity); combined activities of complex I + III (NADH cytochrome c oxidoreductase, rotenone-sensitive activity) and II + III (succinate cytochrome c reductase, malonate–sensitive activity); and CS was determined as described (Spinazzi et al, 2012). On the other hand, complex V (ATP synthase, oligomycin-sensitive activity) activity was measured according to Bénit et al (2006). The measurement was performed in muscle homogenates for skeletal muscles and isolated mitochondria for hiPSC-derived myotubes. Muscle homogenates and myotube mitochondria were subjected to three freeze–thaw cycles to increase rotenone sensitivity. Subsequently, the OxPhos and CS activities were determined spectrophotometrically with Cary 60 UV-Vis spectrometer (Agilent) or Spark Cyto (Tecan). CS activities were used to normalize across samples as an indicator of mitochondrial mass.

### Computational prediction of DD-miRNA targets

Targets of Dlk1-Dio3 miRNAs were predicted by using R package miRNAtap (Pajak, 2007), compiling data from five public databases: DIANA, Targetscan, PicTar, Miranda, and miRDB. The targets used in subsequent analysis are genes predicted by at least two databases.

### Data Availability

The results were presented as mean ± SEM of at least three replicates. PRISM 7.01 program (GraphPad) and R-Studio (version 4.0.3) were used for statistics. Comparisons between two groups were done using *t* test. Comparisons between two groups according to the levels of two categorical variables were done using two-way ANOVA. Comparisons between more than two groups were done using one-way ANOVA followed by Tukey HSD test for multiple comparisons. Significance was defined as *P < 0.05, **P < 0.01, ***P < 0.001.

## Supplementary Material

Reviewer comments
