# Supplementary material for: Dlk1-Dio3 cluster miRNAs regulate mitochondrial functions in the dystrophic muscle in Duchenne muscular dystrophy
Source: Life Sci Alliance. 2022 Oct 20;6(1):e202201506. doi: 10.26508/lsa.202201506 (PMC9585966; doi:10.26508/lsa.202201506)
Supplement: Supplementary file 4 [file LSA-2022-01506_TableS4.docx]

**Supplemental Table 4: List of primers, antibodies, reagents, R packages, and public databases used in the present study.**

|  |  |  |  | |
| --- | --- | --- | --- | --- |
| **qPCR-miRNAs primers** | |  |  | |
| **miRNA** | **Supplier** | **Reference** |  | |
| miR-433-3p | Thermo Fisher | 001028 |  | |
| miR-127-3p | Thermo Fisher | 000452 |  | |
| miR-379-5p | Thermo Fisher | 001138 |  | |
| miR-411-5p | Thermo Fisher | 001610 |  | |
| miR-494-3p | Thermo Fisher | 002365 |  | |
| miR-543-3p | Thermo Fisher | 001298 |  | |
| miR-495-3p | Thermo Fisher | 001663 |  | |
| miR-381-3p | Thermo Fisher | 000571 |  | |
| miR-487b-3p | Thermo Fisher | 001285 |  | |
| miR-431-5p | Thermo Fisher | 001979 |  | |
| miR-134-5p | Thermo Fisher | 001186 |  | |
| miR-370-3p | Thermo Fisher | 002275 |  | |
| miR-299a-5p | Thermo Fisher | 000600 |  | |
| miR-541-5p | Thermo Fisher | 002562 |  | |
| miR-409-3p | Thermo Fisher | 002332 |  | |
| miR-410-3p | Thermo Fisher | 001274 |  | |
| miR-337-5p | Thermo Fisher | 002515 |  | |
| U6 | Thermo Fisher | 001973 |  | |
| miR-93-5p | Thermo Fisher | 001090 |  | |
|  |  |  |  | |
|  | |  |  | |
| **qPCR-GE primers (mmu) SYBR** | |  |  | |
| **Targets** | **Forward** | **Reverse** |  | |
| Meg3 | TTGCACATTTCCTGTGGGAC | AAGCACCATGAGCCACTAGG |  | |
| Rian | CGTGTGTGTGTGTGTGTGGT | GCCAAGGTCTCTACCAGCAG |  | |
| Mirg | GGCAAGGTCTAGGATGGACA | CGCCAGCTTCTGAATACTCC |  | |
| Dlk1 | GACCTGGAGAAAGGCCAGTA | AGGGAGAACCATTGATCACG |  | |
| Sar1b | TTCCTTGGATTGGATAATGCCG | GCCAGCAATAGTAAGCTCTTCTG |  | |
| Bnip3 | TCCTGGGTAGAACTGCACTTC | GCTGGGCATCCAACAGTATTT |  | |
| Hsd17b4 | AGGGGACTTCAAGGGAATTGG | GCCTGCTTCAACTGAATCGTAA |  | |
| Cycs | CCAAATCTCCACGGTCTGTTC | ATCAGGGTATCCTCTCCCCAG |  | |
| Pex19 | GACAGCGAGGCTACTCAGAG | GCCCGACAGATTGAGAGCA |  | |
| Uqcc | TTGCTGGTGCGAGTCCTTAG | GTGTCCGCTCCAACAGTCT |  | |
| Timm44 | CTAGGCAGCGGAATCCAATTT | GCAAGCCTGACAAAAACCCTTT |  | |
| Ndufs1 | AGGATATGTTCGCACAACTGG | TCATGGTAACAGAATCGAGGGA |  | |
| Cs | GGACAATTTTCCAACCAATCTGC | TCGGTTCATTCCCTCTGCATA |  | |
| Gpd2 | GAAGGGGACTATTCTTGTGGGT | GGATGTCAAATTCGGGTGTGT |  | |
| Slc25a20 | GACGAGCCGAAACCCATCAG | AGTCGGACCTTGACCGTGT |  | |
| Rplp0 | CTCCAAGCAGATGCAGCAGA | ATAGCCTTGCGCATCATGGT |  | |
|  |  |  |  | |
| **qPCR-GE primers (hsa)** | |  |  | |
| **Targets** | **Forward** | **Reverse** |  | |
| Ndufs1 | TTAGCAAATCACCCATTGGACTG | CCCCTCTAAAAATCGGCTCCTA |  | |
| Ndufv1 | AGGATGAAGACCGGATTTTCAC | CAGTCACCTCGACTCAGGGA |  | |
| Ndufb4 | ATGTCGTTCCCAAAGTATAAGCC | GAAGCAGGTACTCTCGTTTCAG |  | |
| Ndufa9 | GTCACGTTCTGCCATTACTGC | GGTGGTTGACAACATATCGCC |  | |
| Ndufb2 | GGAGGCCGCCTTTTCAGAA | GGAAGGATCAGGATACGGAAAGT |  | |
| Ndufb11 | CGTCCGCTGGGAATCTAGC | ACGGGGTCCTTGTCATAACCA |  | |
| Ndufs2 | GTCCGATTGCCGATTCAGC | GCTTGGGTACATAACAGCTCC |  | |
| Mt-ND1 | CCACCTCTAGCCTAGCCGTTTA | GGGTCATGATGGCAGGAGTAAT |  | |
| Mt-ND6 | CAAACAATGTTCAACCAGTAACCACTAC | ATATACTACAGCGATGGCTATTGAGGA |  | |
| Sdha | CAAACAGGAACCCGAGGTTTT | CAGCTTGGTAACACATGCTGTAT |  | |
| Shdb | ACAGCTCCCCGTATCAAGAAA | GCATGATCTTCGGAAGGTCAA |  | |
| Sdhc | GAGAAGCTCCAGAGCCTTTTAAAGA | CAACTCCCAGTCCCACTGAAG |  | |
| Sdhd | ATTTCTTCAGGACCGACCTATCC | CAGCCTTGGAGCCAGAATG |  | |
| Uqcrfs1 | CTGAATACCGCCGCCTTGAA | ATGCGACACCCACAGTAGTTA |  | |
| Uqcrc1 | GGGGCACAAGTGCTATTGC | GTTGTCCAGCAGGCTAACC |  | |
| Uqcrb | GGTAAGCAGGCCGTTTCAG | AGGTCCAGTGCCCTCTTAATG |  | |
| Uqcrq | CGCGAGTTTGGGAATCTGAC | TAGTGAAGACGTGCGGATAGG |  | |
| Uqcrc2 | TTCAGCAATTTAGGAACCACCC | GGTCACACTTAATTTGCCACCAA |  | |
| Mt-CYB | ATCACTCGAGACGTAAATTATGGCT | TGAACTAGGTCTGTCCCAATGTATG |  | |
| Cox7b | CTTGGTCAAAAGCGCACTAAATC | AAAATCAGGTGTACGTTTCTGGT |  | |
| Cox5a | ATCCAGTCAGTTCGCTGCTAT | CCAGGCATCTATATCTGGCTTG |  | |
| Cox4l1 | GAGAAAGTCGAGTTGTATCGCA | GCTTCTGCCACATGATAACGA |  | |
| Cox6a1 | AGTTGGTGTGTCCTCGGTTTC | GTGAGAGTCTTCCACATGCGA |  | |
| Cox7a1 | CCGCTTTCAGAACCGAGTG | CCCTTCAGGTACAACGGGA |  | |
| Mt-COI | GACGTAGACACACGAGCATATTTCA | AGGACATAGTGGAAGTGAGCTACAAC |  | |
| Atp5f1 | AGGTCCAGGGGTATTGCAG | TCCTCAGGGATCAGTCCATAAC |  | |
| Atp5e | GTGGCCTACTGGAGACAGG | GGAGTATCGGATGTAGCTGAGT |  | |
| Atp5a1 | GTATTGCCCGCGTACATGG | AGGACATACCCTTTAAGCCTGA |  | |
| Atp5b | CCTGTCAGGGACTATGCGG | TCCTTACTGTGCTCTCACCCA |  | |
| Atp5c1 | TCACCAGGAGACTAAAGTCCATC | TATTTTGCTGCCGCTACCATT |  | |
| Mt-ATP6 | TAGCCATACACAACACTAAAGGACGA | GGGCATTTTTAATCTTAGAGCGAAA |  | |
| Gpd2 | GGCAGTGAAAGGGACGATTCT | GCTGCTTTAACATAGGCCAGGT |  | |
| Bnip3 | CAGGGCTCCTGGGTAGAACT | CTACTCCGTCCAGACTCATGC |  | |
| Slc25a20 | GACCAGCCAAAACCCATCAG | AGAGGGTGACCGACGAACA |  | |
| Sar1b | TACAGTGGTTTCAGCAGTGTG | AGTGGGATGTAATGTTGGGACA |  | |
| Acta1 | GGCATTCACGAGACCACCTAC | CGACATGACGTTGTTGGCATAC |  | |
| Tfam | ATGGCGTTTCTCCGAAGCAT | TCCGCCCTATAAGCATCTTGA |  | |
| Ppargc1a | TCTGAGTCTGTATGGAGTGACAT | CCAAGTCGTTCACATCTAGTTCA |  | |
|  |  |  |  | |
| **qPCR-GE primers (mmu) Taqman** | |  |  | |
| **Targets** | **Supplier** | **Reference** |  | |
| Rtl1 | Thermo Fisher | mm02392620_s1 |  | |
| Myod1 | Thermo Fisher | mm00440387_m1 |  | |
| Myog | Thermo Fisher | mm00446195_g1 |  | |
| Des | Thermo Fisher | hs00157258_m1 |  | |
| Myh1 | Thermo Fisher | mm01332489_m1 |  | |
| Myh2 | Thermo Fisher | mm01332564_m1 |  | |
| Myh4 | Thermo Fisher | mm01332541_m1 |  | |
| Myh7 | Thermo Fisher | mm01319006_g1 |  | |
| Eif4g2 | Thermo Fisher | hs00154952_m1 |  | |
| Usmg5 | Thermo Fisher | hs00910071_g1 |  | |
|  |  |  |  | |
| **Other qPCR primers** | |  |  | |
| **Targets** | **Forward** | **Reverse** |  | |
| mitoDNA(mmu) | CTAGAAACCCCGAAACCAAA | CCAGCTATCACCAAGCTCGT |  | |
| gDNA (mmu) | ATGGGAAGCCGAACATACTG | CAGTCTCAGTGGGGGTGAAT |  | |
| AAV DNA | AGTCCGCCCTGAGCAAAGA | GCTGGAGTTCGTGACCGC |  | |
|  |  |  |  | |
| **Primers for screening IG-KO clones** | |  |  | |
| **Primers** | **Sequences** |  |  | |
| F1 | GGTGAGCTTGCATTCAAAGG |  |  | |
| R1 | GAATGCAATGGTCTGTGGCT |  |  | |
| F2 | CTACACTCACCCTAGCCTCC |  |  | |
| R2 | CGAGAGTCTGGCCTTATGTAC |  |  | |
|  |  |  |  | |
| **Antibodies Western Blot** | |  |  | |
|  | |  |  | |
| **Protein** | **Supplier** | **Reference** | **Dilution** | |
| TET2 | ProteinTech | 21207-1-AP | 1/500 | |
| ACTIN | Sigma | A2066 | 1/250 | |
| SLC25A20 | Abcam | ab82678 | 1/500 | |
| BNIP3 | Abcam | ab10433 | 1/500 | |
| SAR1b | Abcam | ab155278 | 1/1000 | |
| NDUFA9 | Abcam | ab14713 | 1/1000 | |
| SDHA | Abcam | ab14715 | 1/2000 | |
| UQCRC2 | Abcam | ab14745 | 1/1000 | |
| MT-COI | Abcam | ab14705 | 1/1000 | |
| COXIV | Abcam | ab64885 | 1/500 | |
| ATP5A | Abcam | ab14748 | 1/1000 | |
|  |  |  |  | |
| **Antibodies FACS** |  |  |  | |
| **Target** | **Supplier** | **Reference** | **Dilution** | |
| IgG2a/k-APC | BD Biosciences | 553932 | 1/50 | |
| IgG2b/k-APC | BD Biosciences | 553991 | 1/50 | |
| IgG2a/k-PE | BD Biosciences | 553930 | 1/50 | |
| IgG2a/k-Biotin | BD Biosciences | 553928 | 1/50 | |
| CD31-APC | BD Biosciences | 551262 | 1/50 | |
| CD45-APC | BD Biosciences | 559864 | 1/50 | |
| Sca1-PE | BD Biosciences | 553108 | 1/50 | |
| VCAM1-Biotion | BD Biosciences | 553331 | 1/50 | |
| Steptavidin-PE.Cy7 | BD Biosciences | 557598 | 1/400 | |
| FcBlock | BD Biosciences | 553142 | 1/300 | |
| 7AAD | Sigma | A9400-1MG | 1/300 | |
|  |  |  |  | |
| **ISH Probe** |  |  |  | |
| **miRNA** | **Supplier** | **Sequence (5'-3')** | **Reference** | |
| hsa-miR-1-3p | Qiagen |  | 339451 | |
| mmu-miR-127-3p | Qiagen | AGCCAAGCTCAGACGGATCC | YD00615633 | |
| hsa-miR-379-5p | Qiagen | CCTACGTTCCATAGTCTACCA | YD00617025 | |
| Scramble probe | Qiagen | GTGTAACACGTCTATACGCCCA | 163039916 | |
|  |  |  |  | |
| **R packages used in the study** | |  |  | |
| **Package** | **Reference** |  |  | |
| DESeq2 | (Love et al. 2014) |  |  | |
| ReactomePA | (Yu and He 2016) |  |  | |
| biomaRt | (Durinck et al. 2009) |  |  | |
| msigdbr | (Subramanian et al. 2005) |  |  | |
| miRNAtap | (Pajak) |  |  | |
|  |  |  |  | |
| **Public database used in the study** | |  |  | |
| **Identifier** | **Detail** | | |  |
| GSE36257 | miRNA mircoarray in diaphragm muscle of mdx and WT mice | | |  |
| E-MTAB-5955 | miRNA sequencing in quiescent and activated satellite cells from WT mice | | |  |
| GSE64379 | RNA-sequencing of quiescent and activated satellite cells from WT mice | | |  |
| GSE47362 | ChIP-seq of quiescent and activated satellite cells from WT mice | | |  |
| GSE6011 | Microarray of muscle biopsies from young DMD patients and age-matched healthy controls | | |  |
| GSE38417 | Microarray of muscle biopsies from young DMD patients and age-matched healthy controls | | |  |

References

Durinck S, Spellman PT, Birney E, Huber W (2009) Mapping identifiers for the integration of genomic datasets with the R/Bioconductor package biomaRt. Nat Protoc 4: 1184–1191. 10.1038/nprot.2009.97

Subramanian A, Tamayo P, Mootha VK, Mukherjee S, Ebert BL, Gillette MA, Paulovich A, Pomeroy SL, Golub TR, Lander ES, et al (2005) Gene set enrichment analysis: A knowledge-based approach for interpreting genome-wide expression profiles. Proc Natl Acad Sci U S A 102: 15545–15550. 10.1073/pnas.0506580102. [www.pnas.orgcgidoi10.1073pnas.0506580102](http://www.pnas.orgcgidoi10.1073pnas.0506580102).

Yu G, He QY (2016) ReactomePA: An R/Bioconductor package for reactome pathway analysis and visualization. Mol Biosyst 12: 477–479. 10.1039/c5mb00663e
