# Supplementary material for: Dlk1-Dio3 cluster miRNAs regulate mitochondrial functions in the dystrophic muscle in Duchenne muscular dystrophy
Source: Life Sci Alliance. 2022 Oct 20;6(1):e202201506. doi: 10.26508/lsa.202201506 (PMC9585966; doi:10.26508/lsa.202201506)
Supplement: Supplementary file 5 [file LSA-2022-01506_TableS5.docx]

**Supplemental Table 5: List of sgRNAs used in the present study**.

Selection of sgRNAs were done by GPP sgRNA Designer (Doench et al. 2016). Number of off-targets (MM0-3) were further identified by CHOPCHOP (Labun et al. 2019). MM0, -1, -2, -3: number of off-targets with 0, 1, 2, 3 mismatches, respectively.

| **sgRNA** | **sgRNA sequences** | **Target sequences** | **MM0** | **MM1** | **MM2** | **MM3** |
| --- | --- | --- | --- | --- | --- | --- |
| sgIGKO_5end | GAACCCAACUGUGUGUGACA | GAACCCAACTGTGTGTGACAGGG | 1 | 0 | 0 | 12 |
| sgIGKO_3end | GCAUUGAAUGUAUGGCCACG | GCATTGAATGTATGGCCACGTGG | 1 | 0 | 0 | 1 |
