# Supplementary material for: Dlk1-Dio3 cluster miRNAs regulate mitochondrial functions in the dystrophic muscle in Duchenne muscular dystrophy
Source: Life Sci Alliance. 2022 Oct 20;6(1):e202201506. doi: 10.26508/lsa.202201506 (PMC9585966; doi:10.26508/lsa.202201506)
Supplement: Supplementary file 6 [file LSA-2022-01506_TableS6.docx]

**Supplemental Table 6: List of off-targets associated with sgRNAs used in this study**.

Off-targets were identified by CHOPCHOP (Labun et al. 2019). Mismatches of off-target sequences are colored in red.

| **sgRNA** | **Location (hg38/GRCh38)** | **Number of mismatches** | **Sequence (including mismatches)** |
| --- | --- | --- | --- |
| sgIGKO_5end | chr1:1326161 | 3 | **a**AACCCA**t**CTGTGTG**g**GACATGG |
| sgIGKO_5end | chr1:110269095 | 3 | GAACCCA**t**C**a**GT**c**TGTGACAGGG |
| sgIGKO_5end | chr10:48837462 | 3 | GA**g**CC**a**AACTGTG**g**GTGACAGGG |
| sgIGKO_5end | chr10:73645297 | 3 | CCCT**c**TCACACAC**g**GTTGG**t**TTC |
| sgIGKO_5end | chr10:131522611 | 3 | CCTTGTC**c**CACACAG**a**T**a**GGTTC |
| sgIGKO_5end | chr11:17401675 | 3 | GA**g**CCCAACT**t**TGTGTG**t**CAGGG |
| sgIGKO_5end | chr12:16319475 | 3 | **a**AACCCAACTGTGTGT**t**AC**c**TGG |
| sgIGKO_5end | chr2:132513114 | 3 | CCGTGT**g**ACACA**tg**GTTGGGTTC |
| sgIGKO_5end | chr20:60305293 | 3 | CCTTG**ca**ACACACAGTT**t**GGTTC |
| sgIGKO_5end | chr5:123613302 | 3 | CCCTGTCACACA**g**A**c**T**g**GGGTTC |
| sgIGKO_5end | chr6:90122061 | 3 | GAACCC**t**AC**ca**TGTGTGACAAGG |
| sgIGKO_5end | chr8:11759288 | 3 | CCGTGTCACACA**t**AG**ag**GGGTTC |
| sgIGKO_3end | chr1:54241432 | 3 | CCACGTGGC**t**A**g**ACAT**g**CAATGC |
